# Non-fused Phospholes as Fluorescent Probes for Imaging of Lipid Droplets in Living Cells

**DOI:** 10.3389/fchem.2017.00028

**Published:** 2017-04-25

**Authors:** Elisabet Öberg, Hanna Appelqvist, K. Peter R. Nilsson

**Affiliations:** Division of Chemistry, Department of Physics, Chemistry and Biology, Linköping UniversityLinköping, Sweden

**Keywords:** phospholes, fluorescence, cells, imaging, lipid droplets

## Abstract

Molecular tools for fluorescent imaging of specific compartments in cells are essential for understanding the function and activity of cells. Here, we report the synthesis of a series of pyridyl- and thienyl-substituted phospholes and the evaluation of these dyes for fluorescent imaging of cells. The thienyl-substituted phospholes proved to be successful for staining of cultured normal and malignant cells due to their fluorescent properties and low toxicity. Co-staining experiments demonstrated that these probes target lipid droplets, which are, lipid-storage organelles found in the cytosol of nearly all cell types. Our findings confirm that thienyl-substituted phospholes can be utilized as fluorescent tools for vital staining of cells, and we foresee that these fluorescent dyes might be used in studies to unravel the roles that lipid droplets play in cellular physiology and in diseases.

## Introduction

Fluorescence based cellular imaging is an essential technique for visualizing the localization and the dynamics of cellular compartments and molecular processes (Weijer, 2003; Giepmans et al., 2006; Pittet and Weissleder, 2011; Germain et al., 2012). Hence, the development of fluorescent dyes for optical imaging of distinct cellular components is essential. In this regard, conjugated poly- and oligoelectrolytes (CPEs and COEs), optoelectronic materials normally used in classical organic electronics, have emerged as novel tools for fluorescent imaging of distinct cellular elements (Björk et al., 2007; McRae et al., 2008; Pu et al., 2010; Ding et al., 2011; Pu and Liu, 2011; Feng et al., 2012; Li and Liu, 2012; Cieślar-Pobuda et al., 2014; Gwozdzinska et al., 2014; Magnusson et al., 2015). Due to their electronically delocalized conjugated backbones, CPEs and COEs exhibit intrinsic fluorescent characteristics and offer the opportunity to use a variety of fluorescent imaging methods, as well as different modes of detection, such as excitation- and emission spectra, as well as fluorescent decay (Magnusson et al., 2014). In addition, these fluorescent tools also display rather high photo-bleaching thresholds and stability, two parameters that occasionally limit the effectiveness of conventional fluorescent reporter systems, such as fluorophore labeled antibodies or fluorescent proteins, for fluorescent imaging of cells (Medintz et al., 2005).

**Figure 1:**
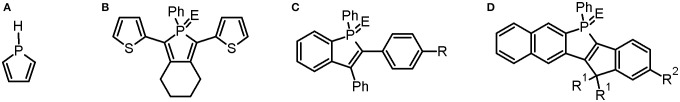
**The chemical structures for different phospholes. (A)** An unsubstituted phosphole. **(B)** A linear phosphole substituted with thienyl groups. E = lone pair, O, S, Se, CH_3_, W(CO)_5_. **(C)** A fused benzophosphole. E = O, S, CH_3_. R = NPh_2_, OMe. **(D)** A fused naphtophosphole. E = O, R^1^ = Ph-4-O-(CH_2_O)_3_-CH_3_, R^2^ = NPh_2_.

**Scheme 1:**
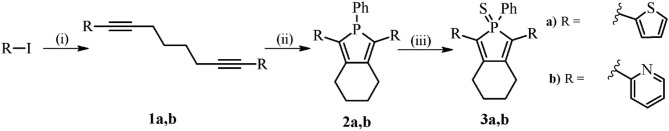
**Synthesis of probes 2–3a,b**. (i) 1,7-octadiyne, Pd(PPh_3_)_2_Cl_2_, CuI, Et_3_N. For **1a**; 2-iodothiophene r.t., 16 h, 87% and **1b**; 2-iodopyridine, 40°C, 16 h, 99%. (ii) Cp_2_ZrCl_2_, *n*-BuLi 2.5 M, −78°C to r.t., 8–16 h, then PhPCl_2_, −78°C to r.t. For **2a**; 40°C, 3 h, 82% and **2b**; r.t., 4–16 h, 62%. (iii) sulfur, DCM, r.t., overnight. For **3a** 40% and **3b** 96%.

From a chemical perspective, the optical and electronic properties of the optoelectronic conjugated materials can be modified by introduction of heteroatoms heavier than carbon, such as sulfur, selenium, or phosphorus, in these systems (Barbarella et al., 2005; Anthony, 2006; Beaujuge and Reynolds, 2010). In this aspect, phosphorus has shown to be especially useful, due to the possibilities to modify the heteroatom through oxidation and metal coordination (Fadhel et al., 2010; Joly et al., 2016; Shameem and Orthaber, 2016). One of the most frequently featured motifs within phosphorus-containing organic conjugated materials is the phosphole moiety. Phospholes are unsaturated five-membered heterocycles analogous to thiophenes and pyrroles. The 1*H*-phosphole, i.e., the unsubstituted phosphole (Figure 1A), was first characterized in 1983 (Charrier et al., 1983). The motif has since then successfully been incorporated in functional materials e.g., as in thienyl-substituted phospholes (Figure 1B). These types of materials have been used in optoelectronic applications such as organic light-emitting diodes (OLEDs), white organic light-emitting diodes (WOLEDs), non-linear optics (NLOs), and solar cells (Crassous and Réau, 2008; Joly et al., 2016; Shameem and Orthaber, 2016). However, in the field of bioimaging, the use of phospholes are relatively scarce and, to the best of our knowledge, there are only two examples to date where benzophospholes (Figure 1C) and napthophospholes (Figure 1D) have been used as fluorescent probes. In these systems, the phosphole ring is fused with a phenyl- or naphtyl-ring. These types of probes have been used for imaging of HeLa cells, adipocytes, and preadipocytes (Wang et al., 2015; Yamaguchi et al., 2015). Herein, we synthesized a series of pyridyl- and thienyl-substituted phospholes (Scheme 1) and evaluated these compounds as fluorescent tools for fluorescent imaging of cells. The thienyl-substituted phospholes could be used for staining of distinct cellular compartments in living cells without major influence on cell viability or proliferation of the cells. In addition, the staining pattern demonstrated high congruence with Nile Red, a fluorescent stain used to stain lipid droplets (Greenspan et al., 1985), dynamic cytoplasmic compartments that are associated with the etiology of several metabolic disorders such as obesity, diabetes and atherosclerosis (Krahmer et al., 2013). Overall, our studies verified that thienyl-substituted phospholes could be utilized for staining of living cells and we foresee that these dyes might be utilized for organelle specific fluorescence based cellular imaging.

## Materials and methods

### General procedures

All synthetic manipulations were carried out under inert atmosphere (N_2_ or Ar) and under ambient conditions, unless otherwise stated. Chemicals were obtained from Sigma-Aldrich and used as received. THF were dried over Na/Benzophenone or 4 Å molecular sieves. ^1^H-NMR and ^13^C-NMR spectra were recorded on a Varian instrument operating at a proton frequency of 300 MHz in CDCl_3_ with or without TMS as internal standard and water. The spectra were referenced to solvent residual peaks as internal standard and reported in ppm (CHCl_3_: δ_H_ = 7.26 ppm, δ_C_ = 77.0 ppm). ^31^P{^1^H}-NMR measurements were recorded on the same instrument. Column chromatography was performed on silica gel, high purity grade, pore size 60 Å, 230–400 mesh particle size. Filtrations were performed on aluminum oxide, activated basic, Brockmann type I. TLC was performed on silica gel matrix plates with a fluorescent indicator at 254 nm from Fluka.

### Synthesis of 1–3a,b

Octadiynes and phospholes **1-3a,b** were prepared according to literature procedures (Hay et al., 2001; Fadhel et al., 2009) and the NMR assignments of the molecules were in line with these reports (see Supplementary Materials).

### Optical characterization of the dyes

Phospholes **2a,b** and **3a,b** were dissolved in DMSO to a final concentration of 1.5 mM. For the emission spectra in DMSO, 7 μl of the stock solution was added to 1000 μl of DMSO yielding a final concentration of 10 μM. For the phosphate buffer saline pH: 7.4 (PBS) solutions, 7 μl of the stock solution was added to 1000 μl of PBS, yielding a final concentration of 10 μM of the probe and 0.7% DMSO. Emission spectra and excitation spectra were collected using a Hitachi U-1900 Spectrophotometer (Hitachi High-Technologies Corporation, Tokyo, Japan) and a Tecan infinite M1000 Pro (Tecan Group Ltd., Männedorf, Switzerland). A smooth function was applied to the excitation and emission data for noise reduction.

### Cell lines and culture conditions

Normal human skin fibroblasts (AG01518; passages 12–24; Coriell Institute, Camden, NJ, USA) and malignant melanoma cells SK-MEL-28 (HTB-72; ATCC, Manassas, VA, USA) were cultured in Eagle's minimum essential medium (EMEM) GlutaMAX, supplemented with 50 IU/ml penicillin-G, 50 μg/ml streptomycin, and 10% fetal bovine serum (all from Gibco, Paisley, UK). Cells were incubated in humidified air with 5% CO_2_ at 37°C. The day before experiments, cells were trypsinized and seeded to reach 50% confluence. For microscopic examination, cells were seeded on glass coverslips No 1.0.

### Vital staining and fixation of cells

The cell lines were stained with 10 μM of **2a,b** and **3a,b** in complete cell culture media for up to 48 h. In all experiments, a corresponding DMSO control was run in parallel. For microscopic evaluation, the cells were rinsed three times with PBS, fixed in 4% paraformaldehyde (PFA; 20 min, 4°C) and mounted using Vectashield without or with DAPI (Vector Laboratories, Burlingame, CA, USA) (Williamson and Fennell, 1975) for visualization of cell nuclei.

### Co-staining with organelle markers

For staining of mitochondria, probe-stained cells were incubated with MitoTracker Orange CMTMRos (150 nM, 30 min, 37°C; Molecular Probes, Eugene, OR). Cells were then fixed in 4% PFA (20 min, 4°C). For immunostaining, stained cells were after fixation permeabilized with 0.1% saponin (Sigma-Aldrich) in PBS containing 5% fetal bovine serum (20 min, room temperature) and incubated for 2 h at room temperature with the monoclonal mouse primary antibodies: lysosome-associated membrane protein 2 (LAMP-2, 1:50; Southern Biotech, Birmingham, AL) or early endosomal antigen-1 (EEA-1, 1:400; Sigma-Aldrich). This step was followed by incubation with the appropriate secondary antibodies conjugated to Alexa Fluor 594 (1:400, Molecular Probes) for 1 h. For staining of lipid droplets fixed cells were stained with 0.1 μg/ml Nile Red in 150 mM NaCl, 10 min at room temperature. All incubations were done in the dark. Next, the cells were mounted in Vectashield (Vector Laboratories) and examined with a Zeiss confocal microscope, LSM 780 (Carl Zeiss AG, Germany).

### Fluorescence microscopy of stained cells

For microscopic analysis of the samples, an inverted LSM 780 confocal microscope (Carl Zeiss, Oberkochen, Germany) was used. Phospholes were generally excited at 405 nm. However, when **2a** and **3a** were used together with DAPI the phospholes were excited at 458 nm or 505 nm. DAPI was excited at 405 nm and emission collected at 410–443 nm, while the emission from the probes were collected at 510–749 nm. A plan-Apochromat 633/1.40 Oil DIC 60 X M27 objective was used for all imaging.

### Viability analysis

The reducing capacity of cell cultures was measured using the 3-(4,5-dimethylthiazol-2-yl)-2,5-diphenyltetrazolium bromide (MTT) reduction assay (Sigma-Aldrich). This method is widely used to assess cytotoxicity and cell viability, and it is currently thought that the amount of MTT formazan is proportional to the number of living cells (van Meerloo et al., 2011). Cells were incubated with 0.5 mg/ml MTT for 2 h at 37°C. Then, the MTT solution was removed and the formazan product was dissolved in DMSO. The absorbance was measured at 550 nm with a VICTOR^TM^ X Series Multiple Plate Reader (PerkinElmer, Waltham, MA). In addition, cell survival was analyzed using the crystal violet assay. Cells were fixed in 4% PFA (20 min, 4°C), stained with 0.04% crystal violet in 1% ethanol (20 min, room temperature), washed extensively with water and thereafter air-dried. The dye was dissolved in 1% SDS (3 h, rocking, room temperature) and thereafter the absorbance was measured at 550 nm.

### Statistical analysis

All experiments were repeated four times and the results are presented as the means and standard deviations of independent samples. Data were statistically evaluated using ANOVA. *P* ≤ 0.05 were considered to be significant. All calculations were done using SPSS Statistics 24.

## Results and discussion

### Synthesis and optical characterization of the phospholes

For the preparation of the phosphole derivatives, two structural motifs were selected, the thienyl, and the pyridyl unit. Pyridyl-substituted phospholes have started to find their way into biological and medicinal chemistry and the idea was to use phosphole **2b** (Scheme 1) as a reference system since it has shown to have a low toxicity in the human breast cancer cell line MCF-7 (Viry et al., 2008). In addition, the thienyl substituent was chosen in order to have a probe with a more red-shifted spectrum compared to the pyridyl-substituted phosphole (Hay et al., 2001). The synthesis of the phospholes is presented in Scheme 1, and was performed in accordance with established procedures (Hay et al., 2001; Fadhel et al., 2009). Firstly, octadiynes **1a** and **1b** were synthesized through a Sonogashira cross-coupling reaction, with Pd(PPh_3_)_2_Cl_2_ and CuI as catalysts in triethyl amine. Secondly, the Fagant-Nugent approach was applied in order to form the phosphole (Fagan and Nugent, 1988; Fagan et al., 1994). In this reaction, a zirconium intermediate is formed, which converts to a phosphole upon addition of *P,P*-dichlorophenylphosphine (Hay et al., 2001; Fadhel et al., 2009). Phospholes **2a,b** were filtered on basic alumina and in contrast to earlier reports, **2a,b** were purified by flash chromatography on silica. This methodology decreased the yield, compared to the earlier reported methods, but was a convenient way to isolate **2a,b** as yellow solids. In the last step, phospholes **2a,b** were oxidized with elemental sulfur and thioxophosphole **3a** was obtained as an orange solid and **3b** as an orange to yellow solid. The structure of the synthesized compounds were confirmed with ^1^H-NMR, ^13^C-NMR, and ^31^P NMR (Supplementary Material).

**Figure 2:**
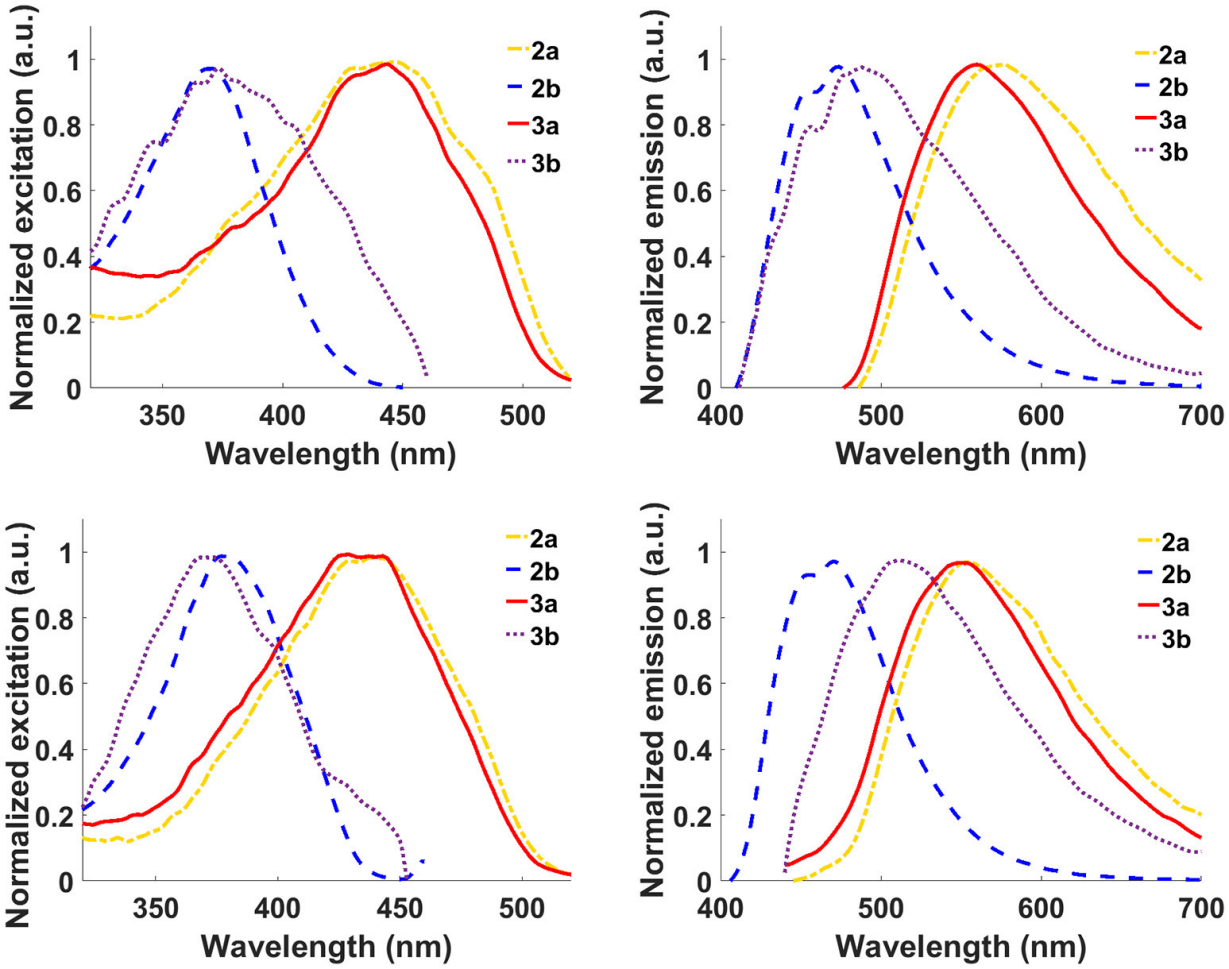
**Optical characterization of the phospholes**. Normalized excitation spectra (left column) and emission spectra (right column) of probes **2a,b** and **3a,b** in phosphate buffer saline pH: 7.4 (PBS) with 0.7% DMSO as co-solvent (top) and DMSO (bottom). Normalization was done with respect to the emission maxima. Ligands **2a** and **3a** were excited at 420 nm and **2b** and **3b** at 390 nm.

Next, the optical properties of the probes in different solvents were explored. For cell imaging using phosphole probes, phosphate buffer saline pH: 7.4 (PBS) with DMSO as co-solvent have been employed earlier (Viry et al., 2008; Wang et al., 2015). Therefore, the excitation- and emission characteristics of the newly synthesized phospholes were measured both in pure DMSO and in PBS with 0.7% DMSO (PBSD) as co-solvent. In DMSO, **2b** and **3b** exhibited excitation maxima at 377 and 374 nm, whereas **2a** and **3a** displayed excitation maxima at longer wavelength, 439 and 429 nm, respectively (Figure 2 and Table 1). In addition, **2a** and **3a**, displayed a red-shift of the excitation maxima in PBSD compared to pure DMSO. The thienyl associated red-shifts were also apparent when comparing the emission characteristic of the phospholes. In DMSO, **2a** and **3a** exhibited emission maxima at 554 and 550 nm, whereas the pyridyl analogs, **2b** and **3b** showed emission maxima at shorter wavelength, 470 and 512 nm, respectively (Figure 2 and Table 1). Thus, in agreement with the chemical design, introduction of the thienyl substituent to the phosphole induced more red-shifted excitation and emission characteristics compared to the previously reported pyridyl moiety (Hay et al., 2001).

**Table 1:** **Excitation- and emission maxima of probes 2a,b and 3a,b in PBSD^a^ and DMSO**.

**Probe**	**Exc_max_ PBSD^a^**	**Em_max_ PBSD^a^**	**Stoke's shift PBSD^a^**	**Exc_max_ DMSO**	**Em_max_ DMSO**	**Stoke's shift DMSO**
2a	446	574	128	439	554	115
2b	371	474	103	377	470	93
3a	443	560	117	429	550	121
3b	374	490	116	374	512	138

a *PBS with 0.7% DMSO*.

### Staining of living cells with the phospholes

**Figure 3:**
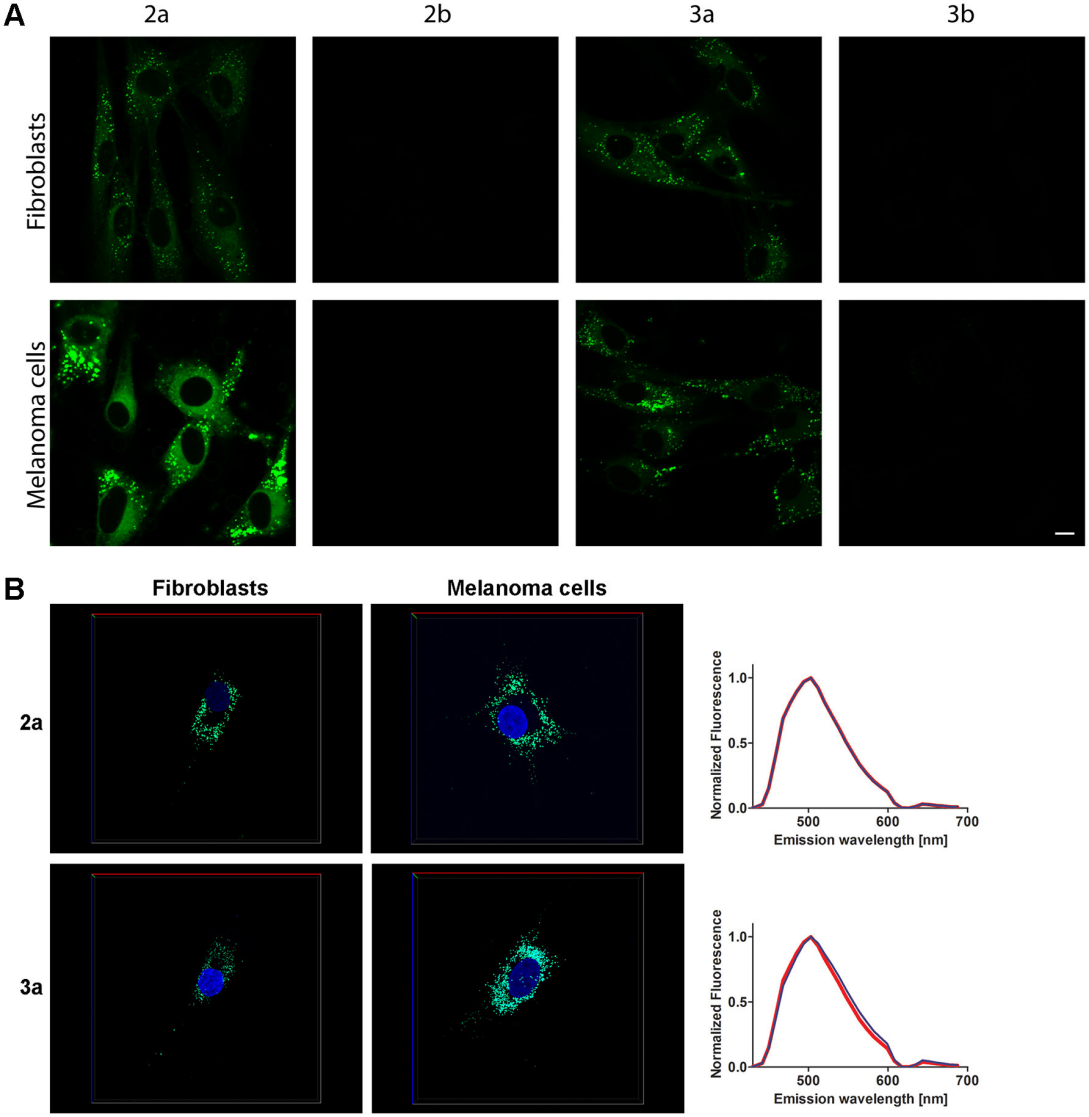
**Live staining of cells with the phosphole ligands. (A)** Fluorescence images of human fibroblasts (AG01518) and malignant melanoma cells (SK-MEL-28) stained with phospholes **2–3a,b**. Living cells were stained with probes (10 μM, 24 h) in complete cell culture medium and thereafter fixated. The cells were analyzed using an excitation wavelength at 405 nm. Scale bar = 10 μm. **(B)** 3D images of AG01518 (left) and SK-MEL-28 (middle) stained by DAPI (blue) and **2a** or **3a**. Images were collected in z-stack spectral mode (excitation at 405 and 458 nm) with the dimensions *x* = 135 μm (red line), *y* = 135 μm (blue line) and *z* = 7.5 μm (AG01518) or 13 μm (SK-MEL-28) (green line). Emission spectra from **2a** or **3a** in the cytoplasmic compartments in AG01518 (blue) and SK-MEL-28 (red). Spectra were collected from five individual cytoplasmic compartments in 20 specific cells for each cell type and the spectra shown in **(B)** are the average of 100 spectra.

Next, the non-fused phospholes were utilized to stain normal human skin fibroblasts (AG01518) and malignant melanoma cells (SK-MEL-28). Living cells were stained with 10 μM phosphole probes in complete cell culture medium for 24 h followed by fixation of the cells with paraformaldehyde. A punctuated intracellular staining pattern was seen with both **2a** and **3a** (Figure 3A). In contrast, the pyridyl-substituted phospholes **2b** and **3b** did not stain the cells to any substantial degree under similar conditions (Figure 3A). However, with increased gain a punctate staining pattern could be observed for **3b** (data not shown). The lack of fluorescent cell staining when using pyridyl-substituted phospholes have also been observed earlier in a cancer cell line (Viry et al., 2008). Hence, our results verified that thienyl-substituted phospholes had a superior performance regarding fluorescence cell staining compared to the pyridyl-substituted phospholes and that the thienyl substituent was necessary for achieving phosphole based ligands for fluorescent imaging of cells. These probes were therefore chosen for further investigations.

Compounds **2a** and **2b** could also be applied together with a conventional nuclear stain, such as DAPI (Figures 3B,C). As shown in Figure 3, the spectra recorded from the thienyl-substituted phospholes stained compartments were similar for both of the dyes and displayed emission maxima at 505 nm. Thus, the spectra obtained from the thienyl-substituted phospholes in this cellular compartments were strikingly blue-shifted compared to the emission maxima obtained from the dyes in PBSD (Figure 2 and Table 1). The blue-shift might indicate that the dyes bind in a cellular compartment having a less polar environment than water, since the emission spectra are blue-shifted for both compound **2a** and **3a** in pure DMSO compared to PBSD (Figure 2).

**Figure 4:**
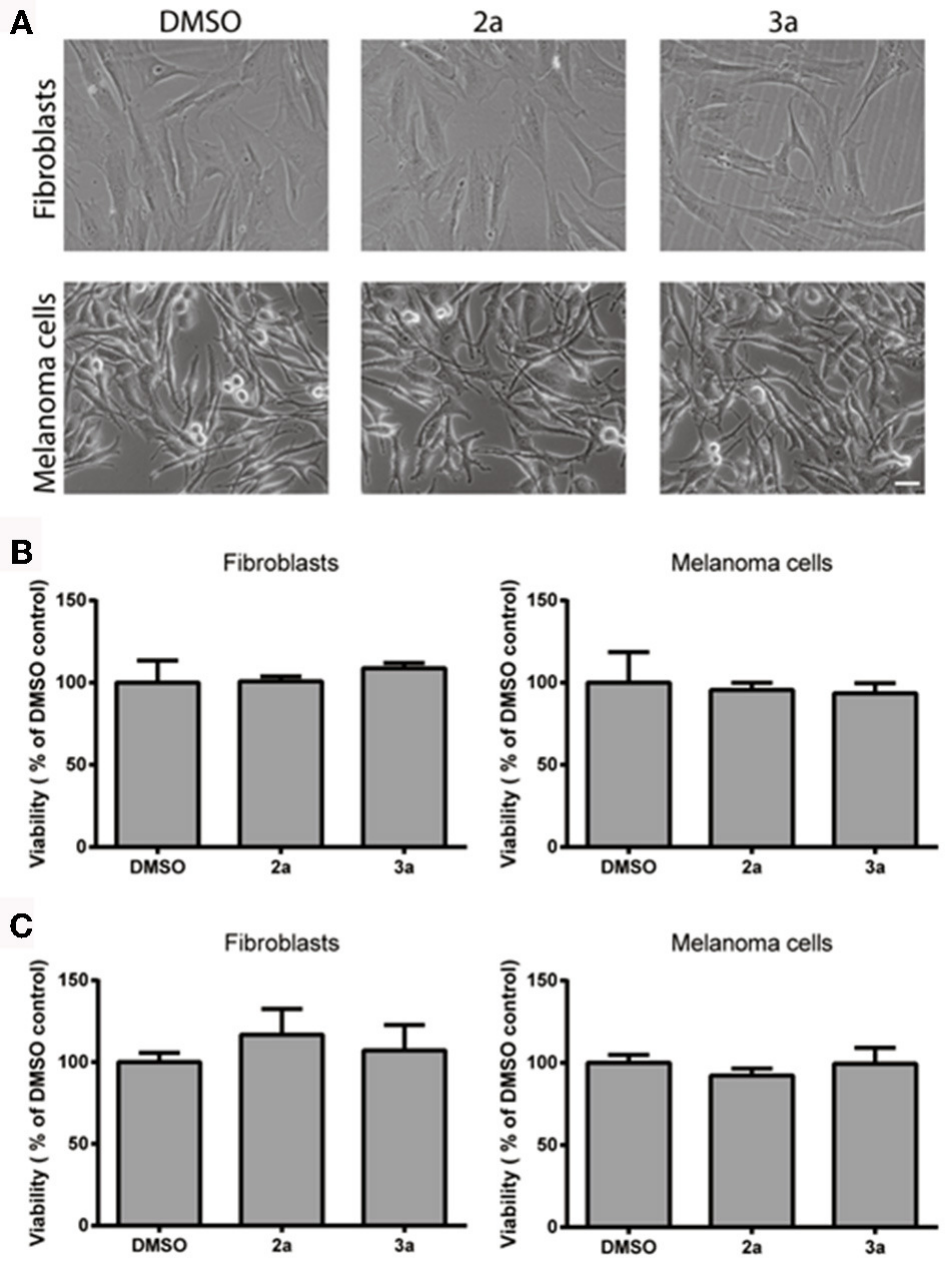
**Cell viability after staining with the thienyl-phospholes. (A)** Phase contrast images of fibroblasts (AG01518) and melanoma cells (SK-MEL-28) after 48 h. Scale bar = 20 μM. **(B)** Cell viability, as determined by the MTT assay **(B)** or the crystal violet assay **(C)**, after 48 h incubation with probes **2, 3a** in fibroblasts and melanoma cells. Data was statistically analyzed with ANOVA and no significant effect on cell viability was found.

For use as vital cell stain, it is essential that the probes do not interfere with cell viability. Therefore the long-term effect of the probes on the viability of stained cultures was investigated using the MTT assay, in which the capacity of the cells to reduce soluble MTT into formazan is a measure of cell viability (Denizot and Lang, 1986). The morphology of stained cells was similar to controls (with corresponding DMSO concentration; Figure 4A) and no significant effect on cell viability was observed in fibroblast or melanoma cell cultures 48 h after staining with **2a** or **3a** (Figure 4B). The results were verified using the crystal violet assay (Figure 4C) and thus a significant toxic effect of these compounds can be excluded. Overall, the cell staining experiments, as well as the cell viability studies, suggested that thienyl-substituted phospholes can be used as fluorescent tools for live imaging of cells.

### Intracellular target of the thienyl-phospholes

**Figure 5:**
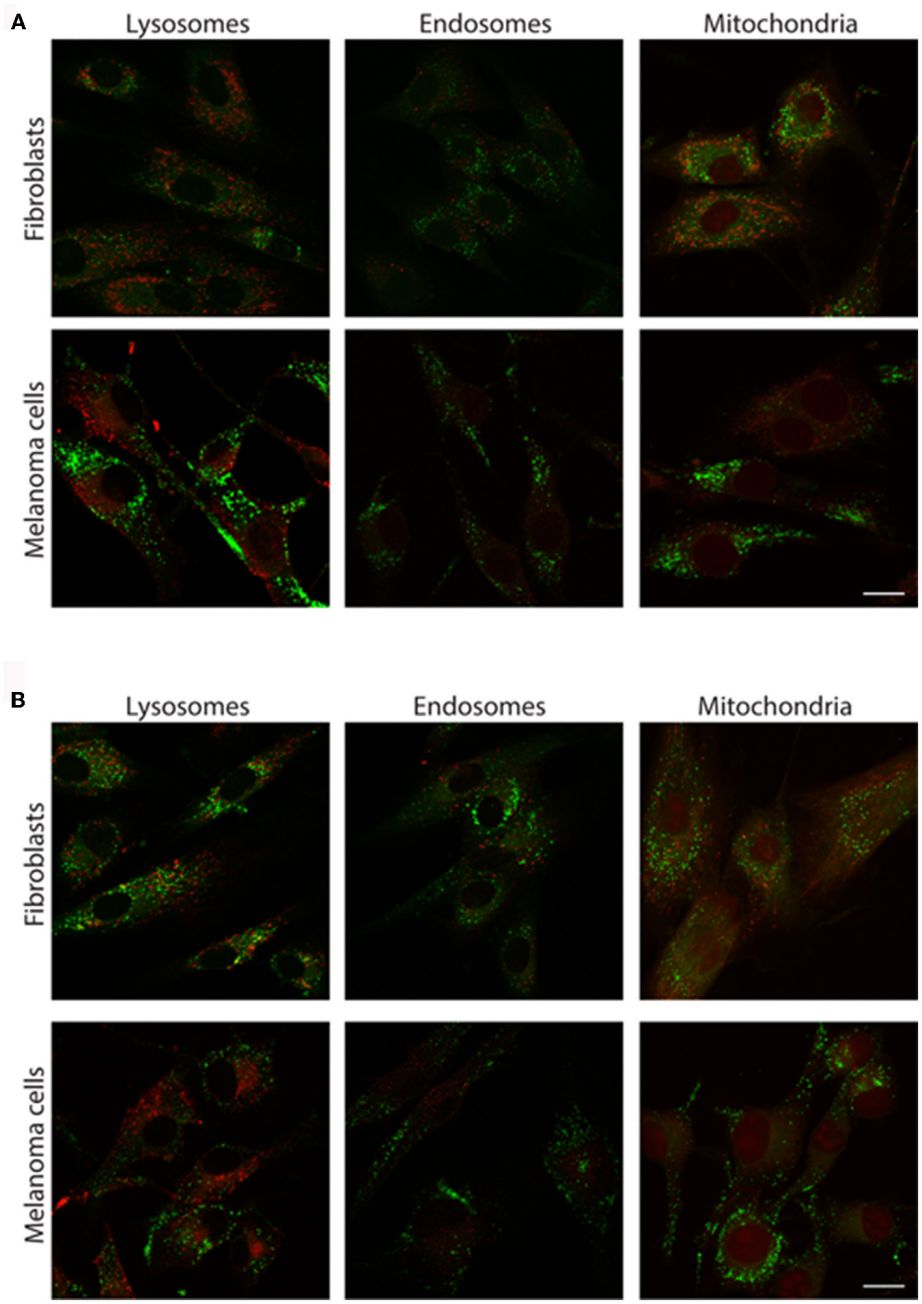
**Costaining of cells with the thienyl-phospholes and markers for intracellular compartments**. Fluorescence images of human fibroblasts (AG01518) and malignant melanoma cells (SK-MEL-28) stained with phospholes **2a (A)** and **3a (B)**, seen in green. The cells were co-stained with markers for lysosomes (LAMP-2), early endosomes (EEA-1), and mitochondria (Mitotracker), seen in red. Scalebars = 10 μm.

As **2a** and **3a** did not influence cell viability and demonstrated a punctuated intracellular cytoplasmic staining pattern, we continued to investigate the potential cellular targets of the thienyl-substituted phospholes. First the phosphole ligands were tested in combination with different conventional fluorescent markers toward major cellular organelles. However, no co-localization was observed with fluorescent markers toward lysosomes, endosomes, or mitochondria (Figure 5), indicating that the thienyl-substituted phospholes stained a different cytoplasmic compartment than these major cellular organelles.

**Figure 6:**
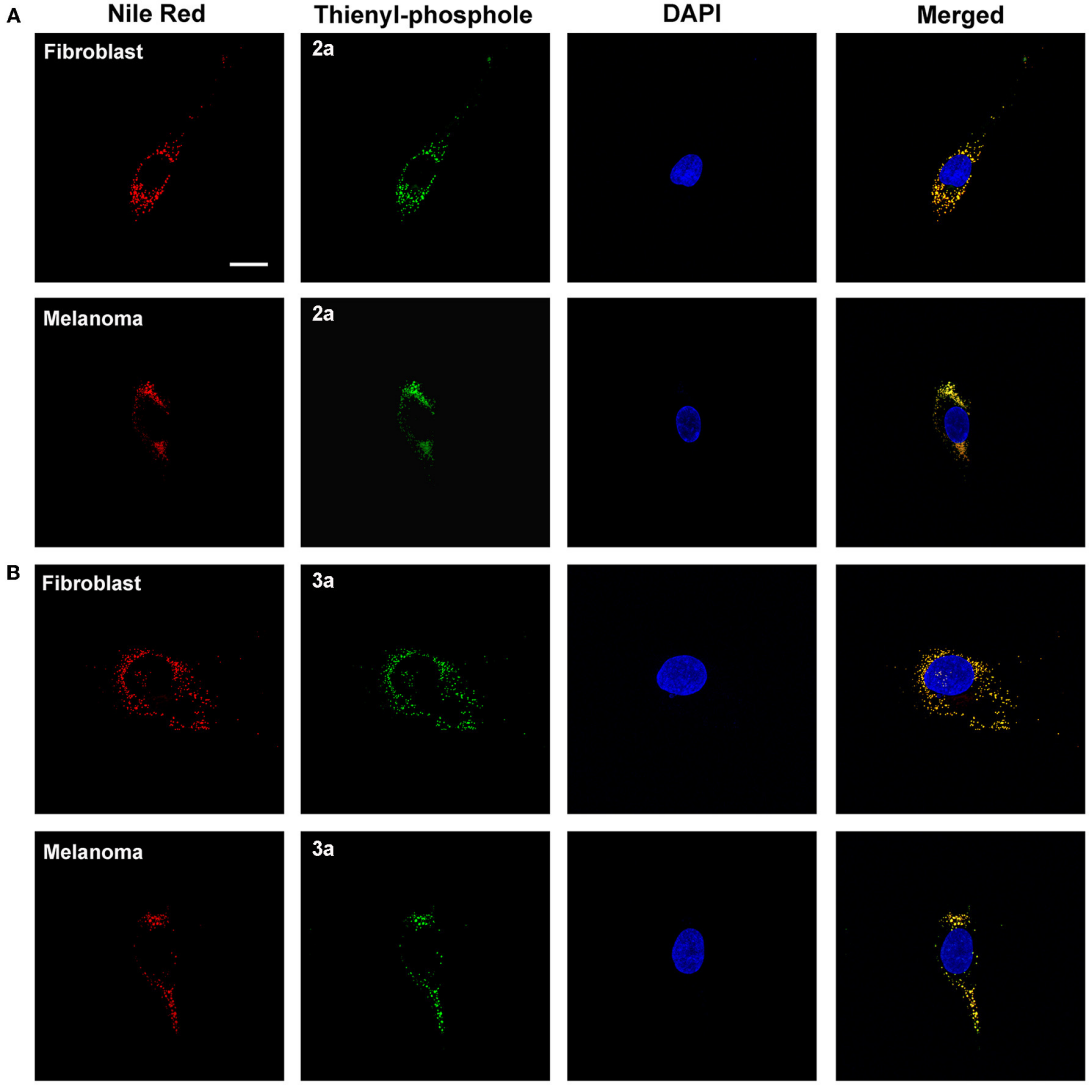
**Intracellular targets of the thienyl-phospholes**. Fluorescence images of human fibroblasts (AG01518) and malignant melanoma cells (SK-MEL-28) stained with phospholes **2a (A)** and **3a (B)** (green, 10 μM, 24 h). After fixation the cells were stained with 0.1 μg/ml Nile Red (red) and the cell nuclei was visualized with DAPI staining (blue). The phospholes displayed a high congruence in staining pattern with Nile Red, as seen as yellow in merged images, indicating that the phospholes stain lipid droplets. Images were collected in z-stack mode (excitation at 405 and 535 nm) with the dimensions *x* = 135 μm, *y* = 135 μm, and *z* = 7.5 μm (AG01518) or 13 μm (SK-MEL-28). Scale bar = 10 μm.

Small hydrophobic dyes, such as Nile Red (Greenspan et al., 1985), BODIPY dyes (Spandl et al., 2009), fluoranthenes (Goel et al., 2014), monodansylpentane (Chen et al., 2017), azafluorenes, and azafluorenone derivatives (Sharma et al., 2016), have previously been utilized for selective fluorescent staining of intracellular lipid droplets. Lipid droplets are found in the cytosol of most eukaryotic cells, where they function as energy reservoirs, sources of lipids for membrane biosynthesis and storage sites for lipids (Farese and Walther, 2009; Beller et al., 2010). In addition, lipid droplets have also been shown to be a key cellular organelle in metabolic disorders, such as diabetes and obesity, as well as in cancer and inflammation (Bozza and Viola, 2010; Greenberg et al., 2011). Hence, fluorescent markers identifying these cytoplasmic compartments are of great interest. Interestingly, both **2a** and **3a** showed a high congruence in staining pattern with Nile Red (Figure 6). Thus, these results indicated that the thienyl-substituted phospholes presented herein can be used for fluorescent assessment of lipid droplets.

## Conclusions

In conclusion, a series of pyridyl- and thienyl-substituted phospholes was synthesized. Fluorescence studies show that the probes exhibit different emission maxima depending on the polarity of the surrounding environment. In addition, this report shows the first example of staining of cultured cells with non-fused phospholes. Thienyl-substituted phospholes **2a** and **3a** show a punctuated staining pattern and co-staining experiments revealed that this pattern is congruent with the staining pattern of Nile Red. Thus, the thienyl-substituted phospholes are therefore potential probes to visualize lipid droplets and for studying the mechanism affecting these structures. We foresee that these fluorescent dyes might aid in studies to unravel the roles that lipid droplets play in cellular physiology and in diseases.

## Author contributions

EÖ, HA, and KPRN designed and performed the experiments, and wrote the manuscript.

### Conflict of interest statement

The authors declare that the research was conducted in the absence of any commercial or financial relationships that could be construed as a potential conflict of interest.

## References

[B1] Anthony J. E. (2006). Functionalized acenes and heteroacenes for organic electronics. Chem. Rev. 106, 5028–5048. 10.1021/cr050966z 17165682

[B2] Barbarella G. Melucci M. Sotgiu G. (2005). The versatile thiophene: an overview of recent research on thiophene-based materials. Adv. Mater. 17, 1581–1593. 10.1002/adma.200402020

[B3] Beaujuge P. M. Reynolds J. R. (2010). Color control in n-conjugated organic polymers for use in electrochromic devices. Chem. Rev. 110, 268–320. 10.1021/cr900129a 20070115

[B4] Beller M. Thiel K. Thul P. J. Jäckle H. (2010). Lipid droplets: a dynamic organelle moves into focus. FEBS Lett. 584, 2176–2182. 10.1016/j.febslet.2010.03.022 20303960

[B5] Björk P. Nilsson K. P. R. Lenner L. Kågedal B. Persson B. Inganäs O.. (2007). Conjugated polythiophene probes target lysosome-related acidic vacuoles in cultured primary cells. Mol. Cell. Probes 21, 329–337. 10.1016/j.mcp.2007.04.005 17553666

[B6] Bozza P. T. Viola J. P. (2010). Lipid droplets in inflammation and cancer. Prostaglandins Leukot. Essent. Fatty Acids 82, 243–250. 10.1016/j.plefa.2010.02.005 20206487

[B7] Charrier C. Bonnard H. De Lauzon G. Mathey F. (1983). Proton [1,5] shifts in P-unsubstituted 1H-phospholes. Synthesis and chemistry of 2H-phosphole dimers. J. Am. Chem. Soc. 105, 6871–6877. 10.1021/ja00361a022

[B8] Chen B. H. Yang H. J. Chou H. Y. Chen C. G. Yang W. Y. (2017). Staining of lipid droplets with monodansylpentane. Methods Mol. Biol. 1560, 231–236. 10.1007/978-1-4939-6788-9_17 28155158

[B9] Cieślar-Pobuda A. Bäck M. Magnusson K. Jain M. V. Rafat M. Ghavami S.. (2014). Cell type related differences in staining with pentameric thiophene derivatives. Cytometry A 85, 628–635. 10.1002/cyto.a.22437 24500794

[B10] Crassous J. Réau R. (2008). π-Conjugated phosphole derivatives: synthesis, optoelectronic functions and coordination chemistry. Dalton Trans. 6865–6876. 10.1039/b810976a 19050770

[B11] Denizot F. Lang R. (1986). Rapid colorimetric assay for cell growth and survival. Modifications to the tetrazolium dye procedure giving improved sensitivity and reliability. J. Immunol. Methods 89, 271–277. 10.1016/0022-1759(86)90368-6 3486233

[B12] Ding D. Pu K. Y. Li K. Liu B. (2011). Conjugated oligoelectrolyte-polyhedral oligomeric silsesquioxane loaded pH-responsive nanoparticles for targeted fluorescence imaging of cancer cell nucleus. Chem. Commun. 47, 9837–9839. 10.1039/c1cc13237g 21808781

[B13] Fadhel O. Benkö Z. Gras M. Deborde V. Joly D. Lescop C.. (2010). 3,4-Dithiaphosphole and 3,3′,4,4′-tetrathia-1,1′-biphosphole π-conjugated systems: S makes the impact. Chem. Eur. J. 16, 11340–11356. 10.1002/chem.201001463 20715213

[B14] Fadhel O. Gras M. Lemaitre N. Deborde V. Hissler M. Geffroy B. (2009). Tunable organophosphorus dopants for bright white organic light-emitting diodes with simple structures. Adv. Mater. 21, 1261–1265. 10.1002/adma.200801913

[B15] Fagan P. J. Nugent W. A. (1988). Synthesis of main group heterocycles by metallacycle transfer from zirconium. J. Am. Chem. Soc. 110, 2310–2312. 10.1021/ja00215a057

[B16] Fagan P. J. Nugent W. A. Calabrese J. C. (1994). Metallacycle transfer from zirconium to main group elements: a versatile synthesis of heterocycles. J. Am. Chem. Soc. 116, 1880–1889. 10.1021/ja00084a031

[B17] Farese R. V. Walther T. C. (2009). Lipid droplets finally get a little R-E-S-P-E-C-T. Cell 25, 855–860. 10.1016/j.cell.2009.11.005 PMC3097139 19945371

[B18] Feng G. Ding D. Liu B. (2012). Fluorescence bioimaging with conjugated polyelectrolytes. Nanoscale 4, 6150–6165. 10.1039/C2NR31392H 22964921

[B19] Germain R. N. Robey E. A. Cahalan M. D. (2012). A decade of imaging cellular motility and interaction dynamics in the immune system. Science 336, 1676–1681. 10.1126/science.1221063 22745423 PMC3405774

[B20] Giepmans B. N. Adams S. R. Ellisman M. H. Tsien R. Y. (2006). The fluorescent toolbox for assessing protein location and function. Science 312, 217–224. 10.1126/science.1124618 16614209

[B21] Goel A. Sharma A. Kathuria M. Bhattacharjee A. Verma A. Mishra P. R.. (2014). New fluoranthene FLUN-550 as a fluorescent probe for selective staining and quantification of intracellular lipid droplets. Org. Lett. 16, 756−759. 10.1021/ol403470d 24410145

[B22] Greenberg A. S. Coleman R. A. Kraemer F. B. McManaman J. L. Obin M. S. Puri V.. (2011). The role of lipid droplets in metabolic disease in rodents and humans. J. Clin. Invest. 121, 2102–2110. 10.1172/JCI46069 21633178 PMC3104768

[B23] Greenspan P. Mayer E. P. Fowler S. D. (1985). Nile red: a selective fluorescent stain for intracellular lipid droplets. J. Cell Biol. 100, 965–973. 3972906 10.1083/jcb.100.3.965 PMC2113505

[B24] Gwozdzinska P. Pawlowska R. Milczarek J. Garner L. E. Thomas A. W. Bazan G. C.. (2014). Phenylenevinylene conjugated oligoelectrolytes as fluorescent dyes for mammalian cell imaging. Chem. Commun. 50, 14859–14861. 10.1039/c4cc06478j 25322778

[B25] Hay C. Hissler M. Fischmeister C. Rault-Berthelot J. Toupet L. Nyulászi L.. (2001). Phosphole-containing pi-conjugated systems: from model molecules to polymer films on electrodes. Chem. Euro. J. 7, 4222–4236. 10.1002/1521-3765(20011001)7:19<4222::AID-CHEM4222>3.0.CO;2-3 11686602

[B26] Joly D. Bouit P. A. Hissler M. (2016). Organophosphorus derivatives for electronic devices. J. Mater. Chem. C 4, 3686–3698. 10.1039/C6TC00590J

[B27] Krahmer N. Farese R. V. Jr. Walther T. C. (2013). Balancing the fat: lipid droplets and human disease. EMBO Mol. Med. 5, 973–983. 10.1002/emmm.201100671 23740690 PMC3721468

[B28] Li K. Liu B. (2012). Polymer encapsulated conjugated polymer nanoparticles for fluorescence bioimaging. J. Mater. Chem. 22, 1257–1264. 10.1039/C1JM14397B

[B29] Magnusson K. Appelqvist H. Cieślar-Pobuda A. Wigenius J. Karlsson T. Łos M. J.. (2015). Differential vital staining of normal fibroblasts and melanoma cells by an anionic conjugated polyelectrolyte. Cytometry A 87, 262–272. 10.1002/cyto.a.22627 25605326

[B30] Magnusson K. Simon R. Sjölander D. Sigurdson C. J. Hammarström P. Nilsson K. P. R. (2014). Multimodal fluorescence microscopy of prion strain specific PrP deposits stained by thiophene-based amyloid ligands. Prion 8, 319–329. 10.4161/pri.29239 25495506 PMC4601348

[B31] McRae R. L. Phillips R. L. Kim I.-B. Bunz U. H. F. Fahrni C. J. (2008). Molecular recognition based on low-affinity polyvalent interactions: selective binding of a carboxylated polymer to fibronectin fibrils of live fibroblast cells. J. Am. Chem. Soc. 130, 7851–7853. 10.1021/ja8007402 18507462

[B32] Medintz I. L. Uyeda H. T. Goldman E. R. Mattoussi H. (2005). Quantum dot bioconjugates for imaging, labelling and sensing. Nat. Mater. 4, 435–446. 10.1038/nmat1390 15928695

[B33] Pittet M. J. Weissleder R. (2011). Intravital imaging. Cell 147, 983–991. 10.1016/j.cell.2011.11.004 22118457 PMC3824153

[B34] Pu K. Y. Li K. Zhang X. H. Liu B. (2010). Conjugated oligoelectrolyte harnessed polyhedral oligomeric silsesquioxane as light-up hybrid nanodot for two-photon fluorescence imaging of cellular nucleus. Adv. Mater. 22, 4186–4189. 10.1002/adma.201001544 20589775

[B35] Pu K. Y. Liu B. (2011). Fluorescent conjugated polyelectrolytes for bioimaging. Adv. Funct. Mater. 21, 3408–3423. 10.1002/adfm.201101153

[B36] Shameem M. A. Orthaber A. (2016). Organophosphorus compounds in organic electronics. Chem. Eur. J. 22, 10718–10735. 10.1002/chem.201600005 27276233

[B37] Sharma A. Umar S. Kar P. Singh K. Sachdev M. Goel A. (2016). A new type of biocompatible fluorescent probe AFN for fixed and live cell imaging of intracellular lipid droplets. Analyst 141, 137–143. 10.1039/c5an01623a 26528832

[B38] Spandl J. White D. J. Peychl J. Thiele C. (2009). Live cell multicolor imaging of lipid droplets with a new dye, LD540. Traffic 10, 1579–1584. 10.1111/j.1600-0854.2009.00980.x 19765264

[B39] van Meerloo J. Kaspers G. J. L. Cloos J. (2011). Cell sensitivity assays: the MTT assay. Methods Mol. Biol. 731, 237–245. 10.1007/978-1-61779-080-5_20 21516412

[B40] Viry E. Battaglia E. Deborde V. Müller T. Réau R. Davioud-Charvet E.. (2008). A sugar-modified phosphole gold complex with antiproliferative properties acting as a thioredoxin reductase inhibitor in MCF-7 Cells. Chem. Med. Chem. 3, 1667–1670. 10.1002/cmdc.200800210 18759235

[B41] Wang C. Fukazawa A. Taki M. Sato Y. Higashiyama T. Yamaguchi S. (2015). A phosphole oxide based fluorescent dye with exceptional resistance to photobleaching: a practical tool for continuous imaging in sted microscopy. Angew. Chem. Int. Ed. Engl. 54, 15213–15217. 10.1002/anie.201507939 26493944

[B42] Weijer C. J. (2003). Visualizing signals moving in cells. Science 300, 96–100. 10.1126/science.1082830 12677060

[B43] Williamson D. H. Fennell D. J. (1975). The use of fluorescent DNA-binding agent for detecting and separating yeast mitochondria1 DNA. Methods Cell Biol. 12, 335–351. 1105070 10.1016/s0091-679x(08)60963-2

[B44] Yamaguchi E. Wang C. Fukazawa A. Taki M. Sato Y. Sasaki T.. (2015). Environment-sensitive fluorescent probe: a benzophosphole oxide with an electron-donating substituent. Angew. Chem. Int. Ed. Engl. 54, 4539–4543. 10.1002/anie.201500229 25740735

