# Supplementary material for: Non-fused Phospholes as Fluorescent Probes for Imaging of Lipid Droplets in Living Cells
Source: Front Chem. 2017 Apr 25;5:28. doi: 10.3389/fchem.2017.00028 (PMC5403830; doi:10.3389/fchem.2017.00028)
Supplement: Supplementary file 1 [file DataSheet1.pdf]

Supplementary material

Non-fused phospholes as fluorescent probes for imaging of lipid droplets in living cells

Elisabet Öberg, Hanna Appelqvist, Peter Nilsson

Department of Physics, Chemistry and Biology (IFM), Linköping University, 581 83 Linköping, Sweden

1. **General procedures……………………………………………………………...2**
2. **Synthesis procedures and NMR spectra of 1a,b-3a,b……………….…………...2**
3. **References…………………………………………………………………………14**

General procedures

All synthetic manipulations were carried out under inert atmosphere (N_2 or Ar) and under ambient conditions, unless otherwise stated. Chemicals were obtained from Sigma-Aldrich and used as received. THF were dried over Na/Benzophenone or molecular sieves 4Å. ^{1}H-NMR and ^{13}C-NMR spectra were recorded on a Varian instrument operating at a proton frequency of 300 MHz in $CDCl_3$ with or without TMS as internal standard and water. The spectra were referenced to solvent residual peaks as internal standard and reported in ppm ($CHCl_3$: δ_H = 7.26 ppm, δ_C = 77.0 ppm). ^{31}P{^{1}H}-NMR measurements were recorded on the same instrument. Column chromatography was performed on silica gel, high purity grade, pore size 60 Å, 230-400 mesh particle size. Filtrations were performed on aluminium oxide, activated basic, Brockmann type I. TLC was performed on silica gel matrix plates with a fluorescent indicator at 254 nm from Fluka.

Synthesis procedures and NMR spectra of 1a,b-3a,b.

Octadiynes and phospholes **1-3a,b** were prepared according to literature procedures (Hay et al., 2001; Fadhel et al., 2009) and the NMR assignments are in line with these reports. Description of the specified procedures and analytical data are shown below.

Synthesis of 2,2’-octa-1,7-diyne-1,8-diyldithiophene (1a): $Pd(PPh_3)_2Cl_2$ (400 mg, 0.57 mmol) and CuI (219 mg, 1.1 mmol) were added to 40 ml Et_3N. 1,7-octadiyne (2.5 g, 3.1 ml, 23 mmol) and 2-iodothiophene (7 ml, 63 mmol) were added. A brown precipitate was formed and the slurry was stirred for 16 h. The solvent was removed under vacuum, the residue redissolved in Et_2O and evaporated on silica. Column chromatography was performed in heptane. $R_f = 0.1$. After evaporation 2,2’-octa-1,7-diyne-1,8-diyldithiophene was isolated as a white solid. Yield 5.47 g (20 mmol, 87 %). ^{1}H-NMR ($CDCl_3$, 300 MHz): δ 1.71-1.81 (m, 4H, CH_2), 2 .45-2.54 (m, 4H, CH_2), 6.91-6.96 (m, 2H, CH, thienyl), 7.11-7.14 (m, 2H, CH, thienyl), 7.15-7.19 (m, 2H, CH, thienyl). ^{13}C-NMR ($CDCl_3$, 75.5 MHz): δ 19.3 (CH_2), 27.7 (CH_2), 74.1 (C≡), 93.8 (C≡), 124.1 (C, thienyl), 125.9 (CH, thienyl), 126.8 (CH, thienyl), 131.0 (CH, thienyl).

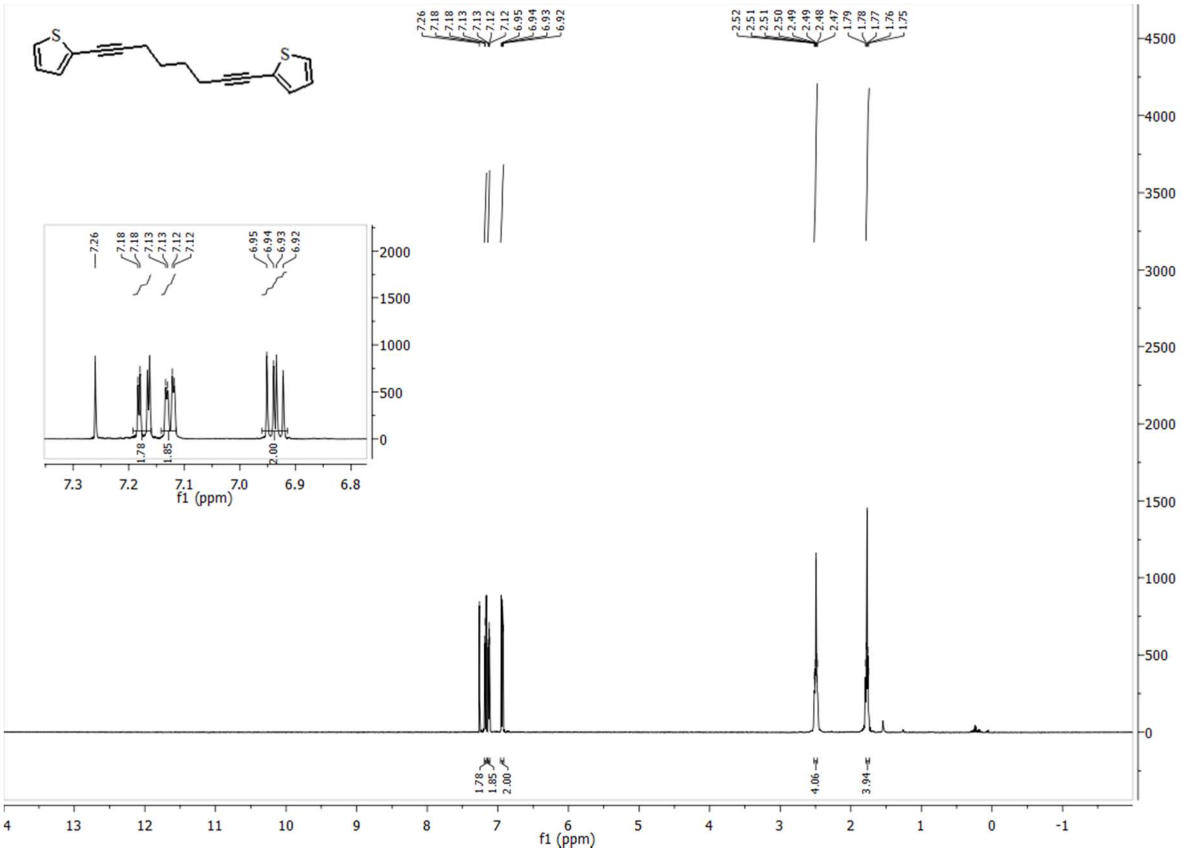

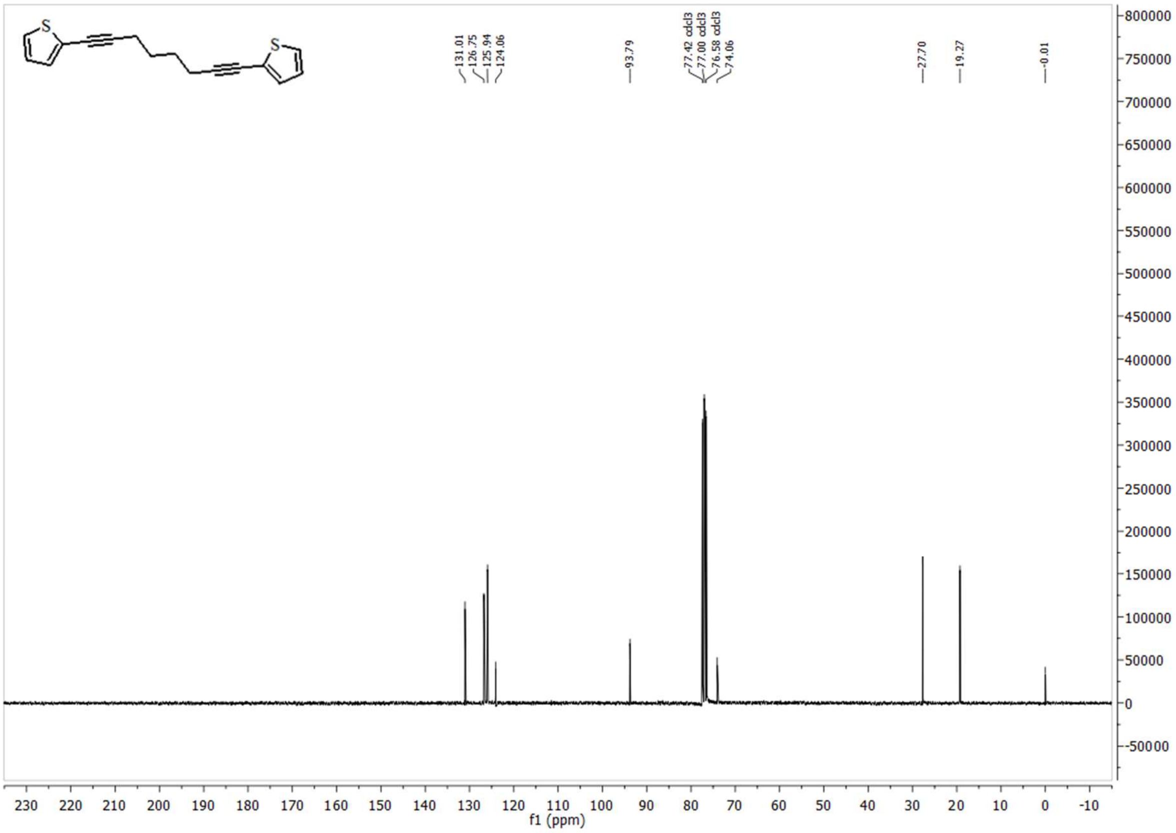

Synthesis of 2,2'-octa-1,7-diyne-1,8-diyldipyridine (1b): $Pd(PPh_3)_2Cl_2$ (210 mg, 0.30 mmol) and CuI (57 mg, 0.30 mmol) were added to 20 ml Et_3N. 1,7-octadiyne (0.4 ml, 3 mmol) and 2-iodopyridine (2.5 g, 12 mmol) were added. A brown precipitate was formed and the slurry was stirred for 16 h at 40 °C. The slurry was evaporated on silica. Column chromatography was performed with 20 % heptane in EtOAc. $R_f = 0.2$. After evaporation 2,2'-octa-1,7-diyne-1,8-diyldipyridine was isolated as an orangebrown solid. Yield 0.733 g (2.98 mmol, 99 %). ^{1}H-NMR ($CDCl_3$, 300 MHz): δ 1.77-1.88 (m, 4H, CH_2), 2.47-2.56 (m, 4H, CH_2), 7.18 (ddd, 2H, J= 1.2 Hz, 4.9 Hz, 7.6 Hz, pyridyl), 7.37 (dt, 2H, J= 1.0 Hz, 7.9 Hz, pyridyl), 7.60 (td, 2H, J= 1.8 Hz, 7.7 Hz, pyridyl), 8.52-8.56 (m, 2H pyridyl). ^{13}C-NMR ($CDCl_3$, 75.5 MHz): δ 18.9 (CH_2), 27.5 (CH_2), 80.8 (C≡), 90.4 (C≡), 122.3 (CH, pyridyl), 126.8 (CH, pyridyl), 136.0 (CH, pyridyl), 143.9 (C, pyridyl), 149.8 (CH, pyridyl).

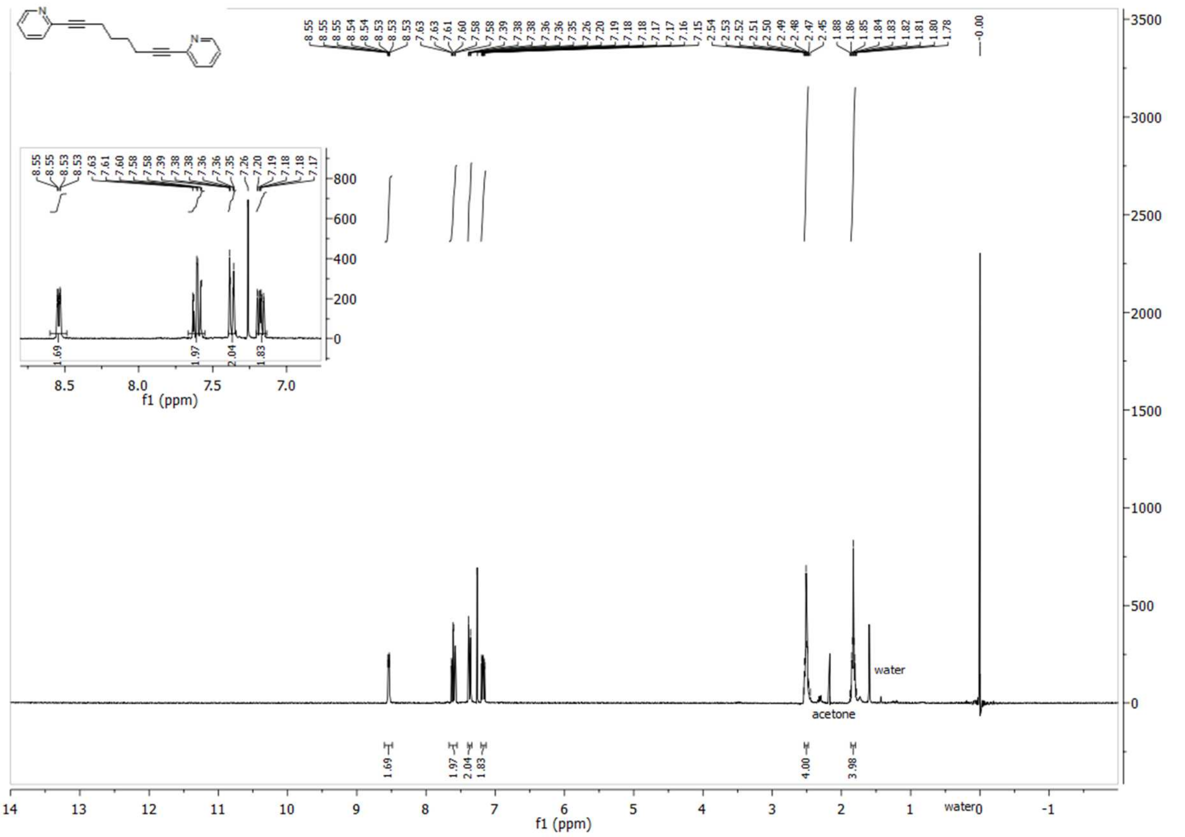
water
acetone
f1 (ppm)

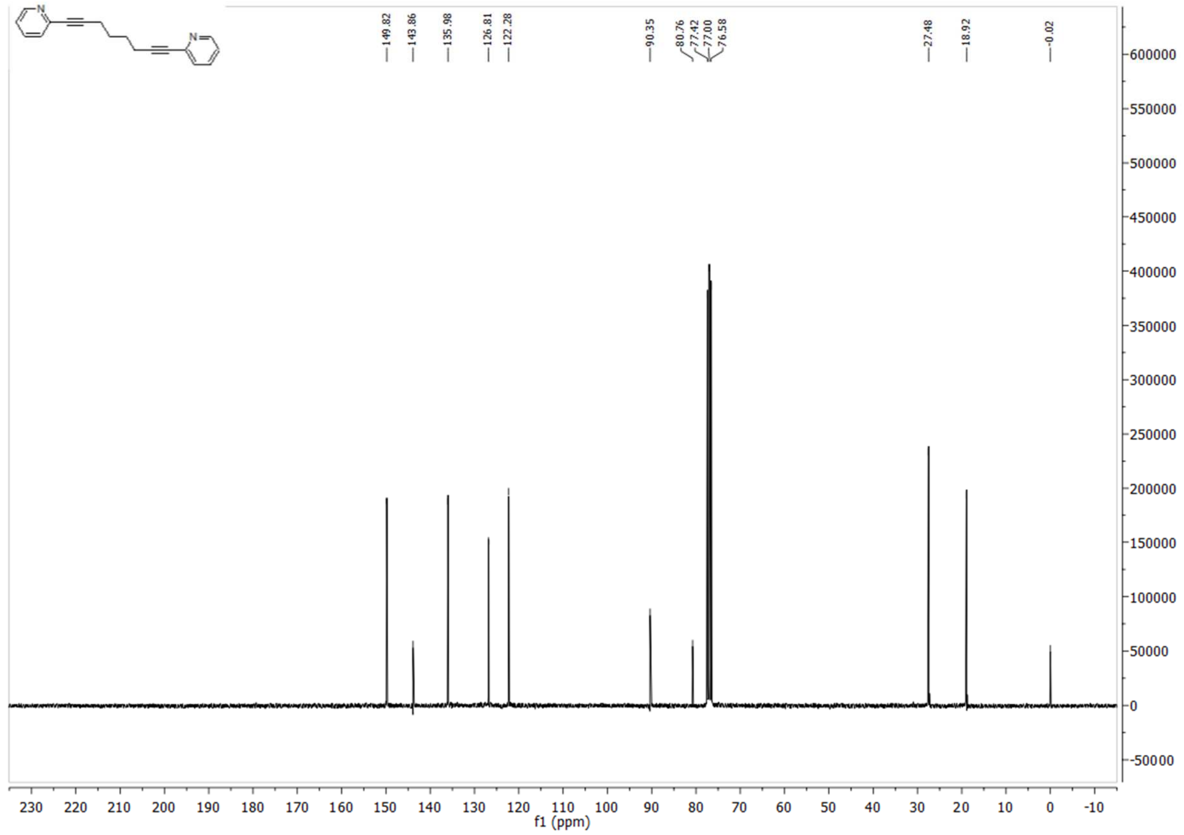
149.82
143.86
135.98
126.81
122.28
90.35
80.76
77.42
77.00
76.58
27.48
18.92
-0.02
f1 (ppm)

Synthesis of Thienyl-phosphole (2a): 2,2'-octa-1,7-diyne-1,8-diyldithiophene (216 mg, 0.80 mmol) and Cp_2ZrCl_2 (250 mg, 0.86 mmol) were dried and THF, 30 ml, was added. The solution was cooled to -78 ^{0}C. *n*-BuLi 2.5 M in hexane (0.70 ml, 1.7 mmol) was added at this temperature and the solution turned dark red upon warming to room temperature. The solution was stirred at room temperature for 16 h. The solution was cooled to -78 ^{0}C and $PhPCl_2$ (0.12 ml, 0.86 mmol) was added. The solution was stirred for 3 h at 40 °C and filtered through alumina. The solution was concentrated, dissolved in CH_2Cl_2 and an excess of sulfur was added. The resulting slurry was filtered and concentrated. Column chromatography with a gradient of heptane to heptane: CH_2Cl_2 10:1 as eluent yields phosphole-thiophene **2a** as a yellow solid. R_f = 0.4. 202 mg (0.66 mmol, 82 %). ^{31}P-NMR ($CDCl_3$, 101 MHz): δ 12.17. ^{1}H-NMR ($CDCl_3$, 300 MHz): δ 1.77-1.88 (m, 4H, CH_2), 2.85-2.93 (m, 4H, CH_2), 6.94 (m, 2H, CH, thienyl), 7.05 (d broad, *J*= 3.6 Hz, 2H, CH, thienyl), 7.18 (dt, *J*= 5.1, 1.2 Hz, 2H, CH, thienyl) overlapping with 7.23 (m, 3H, phenyl), 7.39-7.47 (m, 2H, phenyl). ^{13}C-NMR ($CDCl_3$, 75.5 MHz): δ 23.1 (s, $C{=}CCH_2CH_2$), 29.2 (d, $J_{P,C}$ = 1.5 Hz, $C{=}CCH_2CH_2$), 125.3 (d, , $J_{P,C}$= 9.6 Hz thienyl), 127.2 (s, thienyl), 128.6 (d, $J_{P,C}$= 8.2 Hz, m-C phenyl), 129.6 (d, $J_{P,C}$= 1.6 Hz, p-C phenyl), 133.1 (d, $J_{P,C}$= 12.9 Hz, ipso-C, phenyl), 133.8 (d, $J_{P,C}$= 19.7 Hz, ortho-C phenyl), 135.7 (s, PC=C), 139.5 (d, $J_{P,C}$= 19.7 Hz, thienyl), 144.6 (d, $J_{P,C}$= 8.6 Hz, P=C-C).

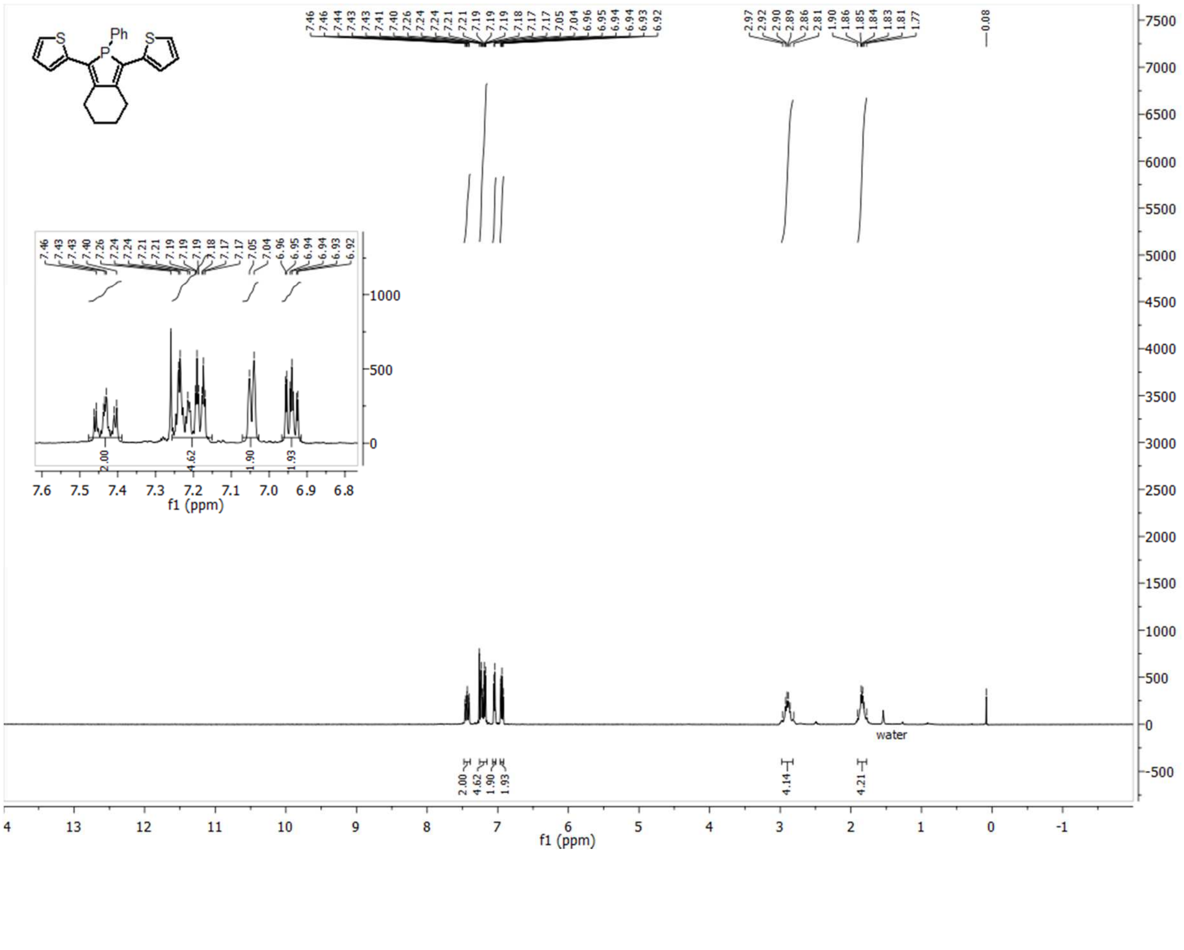

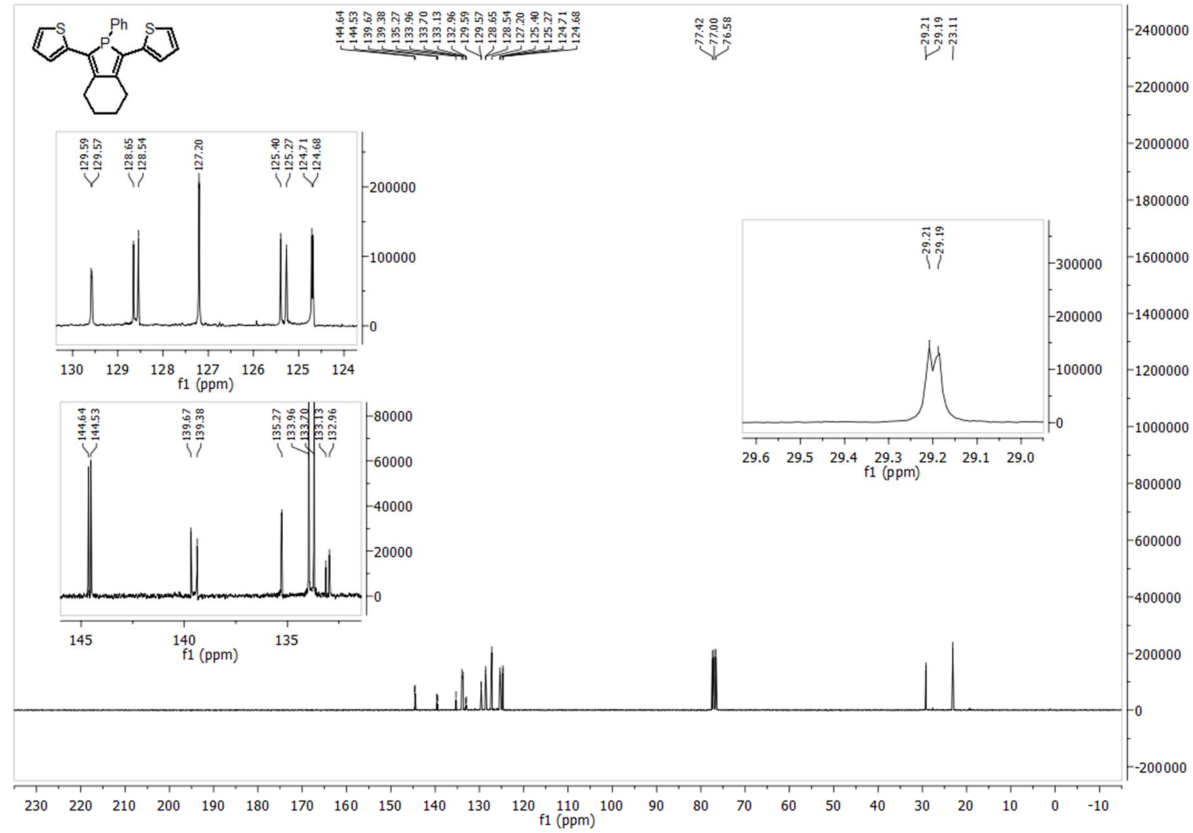

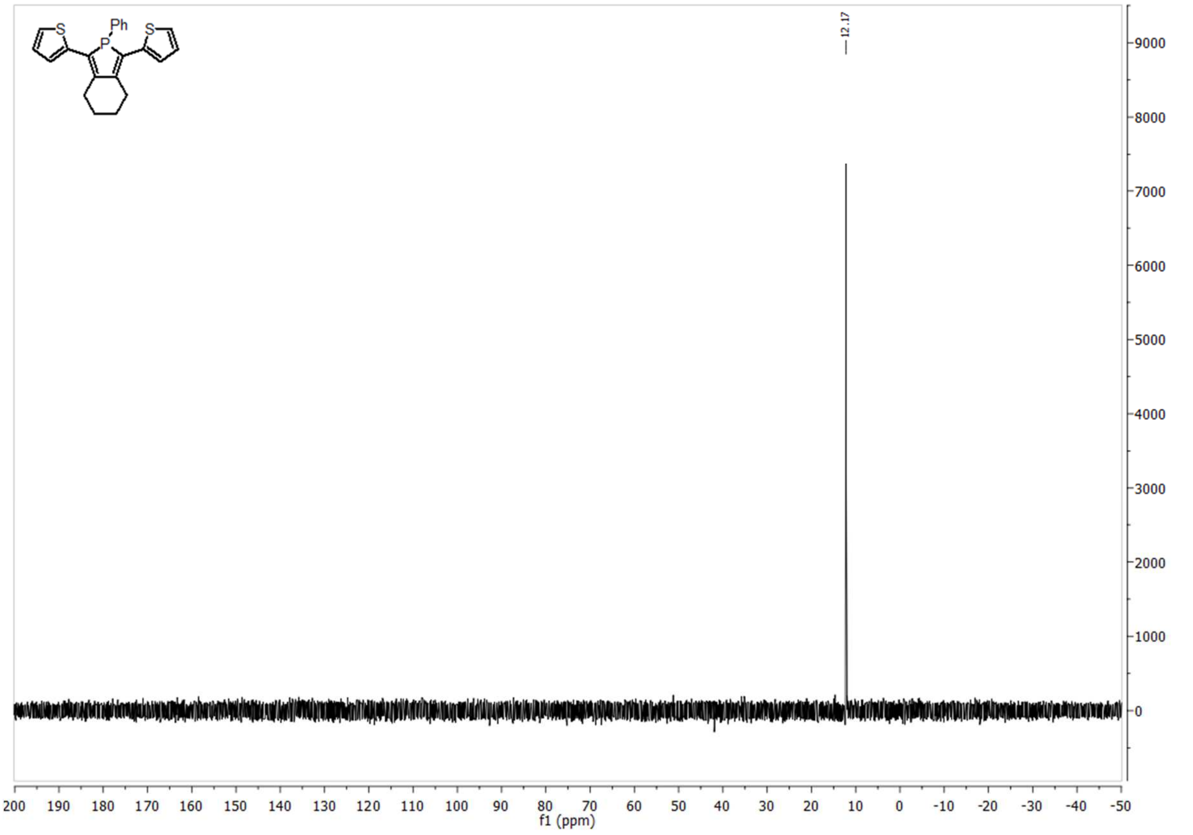

Synthesis of Pyridyl-phosphole (2b): 2,2'-octa-1,7-diyne-1,8-diyldipyridine (350 mg, 1.4 mmol) and Cp_2ZrCl_2 (414 mg, 1.4 mmol) were dried overnight. THF, 20 ml, was added and the solution was cooled to -78 ^{0}C. *n*-BuLi 2.5 M in hexane (1.2 ml, 3.0 mmol) was added at this temperature and the solution turned dark red upon warming to room temperature. The solution was stirred at room temperature for 8 h, cooled to -78 ^{0}C and $PhPCl_2$ (0.20 ml, 1.5 mmol) was added. The solution was stirred for 16 h and filtered through alumina. The solution was concentrated and column chromatography in 1:3 EtOAc:heptane yields **2b** as a yellow solid. R_f = 0.3. 323 mg (0.88 mmol, 62 %). ^{31}P-NMR ($CDCl_3$, 101 MHz): δ 11.47 ^{1}H-NMR ($CDCl_3$, 300 MHz): δ 1.69-1.77 (m, 2H, CH_2), 1.80-1.99 (m, 2H, CH_2), 2.71-2.96 (m, 2H, CH_2) 3.16-3.49 (m, 2H, CH_2), 7.00 (ddd, *J*= 7.3 Hz, 4.9 Hz, 1.3 Hz, 2H, CH, pyridyl), 7.06-7.11 (m, 3H, phenyl), 7.29-7.41 (m, 2H, pyridyl), 7.46 (d broad, *J*= 8.1 Hz, 2H, pyridyl), 7.50-7.60 (m, 2H, phenyl), 8.57 (ddd, *J*= 4.8 Hz, 1.8 Hz, 0.9 Hz, 2H, pyridyl). ^{13}C-NMR ($CDCl_3$, 75.5 MHz): δ 23.1 (s, $C{=}CCH_2CH_2$), 28.8 (s, $C{=}CCH_2CH_2$), 120.5 (s, pyridyl), 123.7 (d, $J_{P,C}$= 9.2 Hz, pyridyl), 128.2 (d, $J_{P,C}$= 8.0 Hz, m-C Ph), 128.9 (d, $J_{P,C}$= 1.5 Hz,para-phenyl), 132.1 (d, $J_{P,C}$= 12.7 Hz,ipso-C- phenyl), 133.8 (d, $J_{P,C}$= 13.8 Hz, ortho-C- phenyl), 135.9 (s, pyridyl), 144.3 (d, $J_{P,C}$= 4.0 Hz, P=C-C),148.1 (d, $J_{P,C}$= 10.3 Hz, P=C-C), 149.3 (d, $J_{P,C}$= 0.9 Hz, pyridyl), 155.8 (d, $J_{P,C}$= 19.3 Hz, pyridyl).

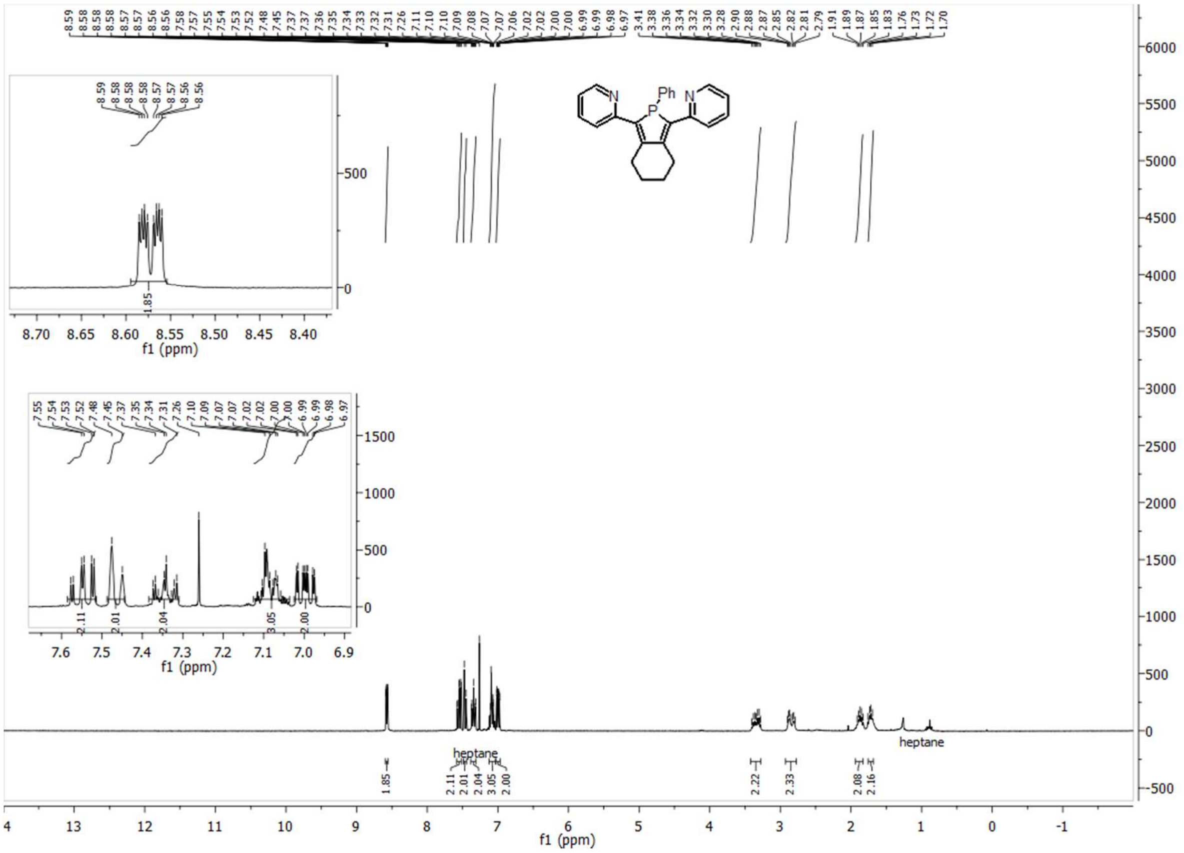

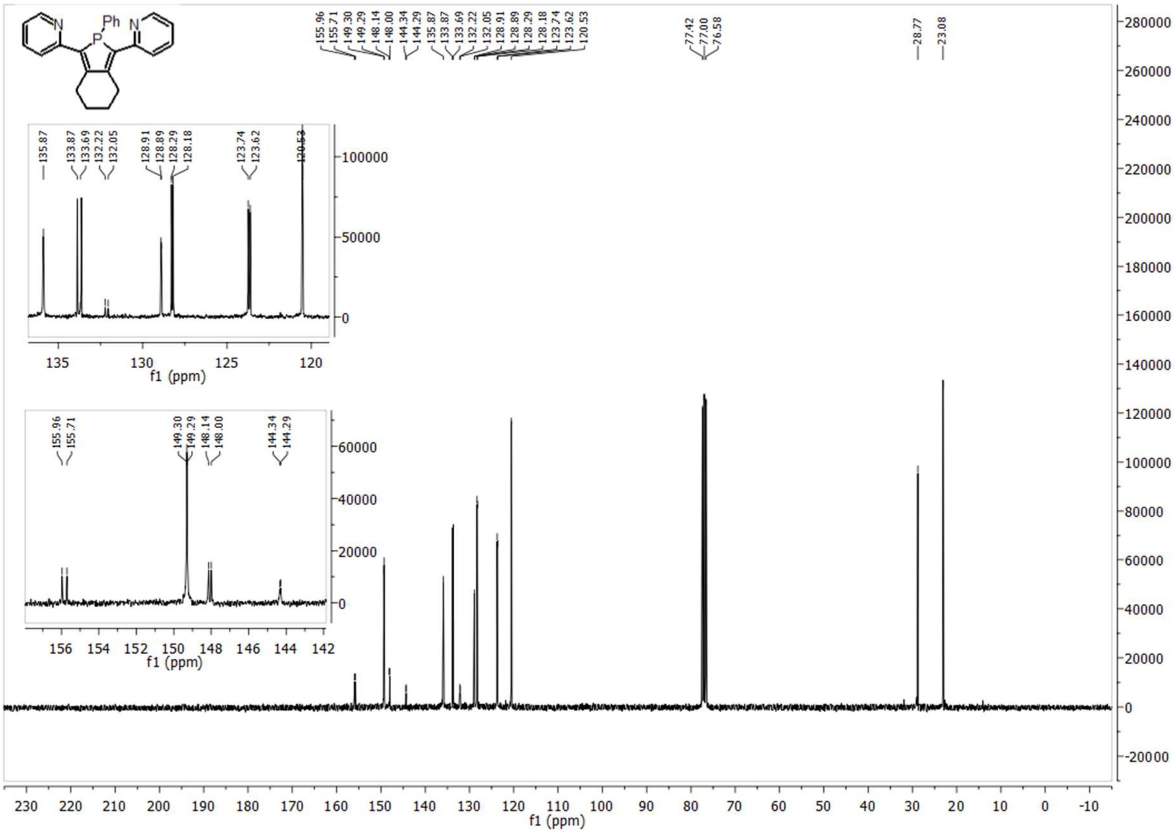
Ph
P
N
155.96
155.71
149.30
149.29
148.14
148.00
144.34
144.29
135.87
133.87
133.69
132.22
132.05
128.91
128.89
128.29
128.18
123.74
123.62
120.53
77.42
77.00
76.58
28.77
23.08
f1 (ppm)

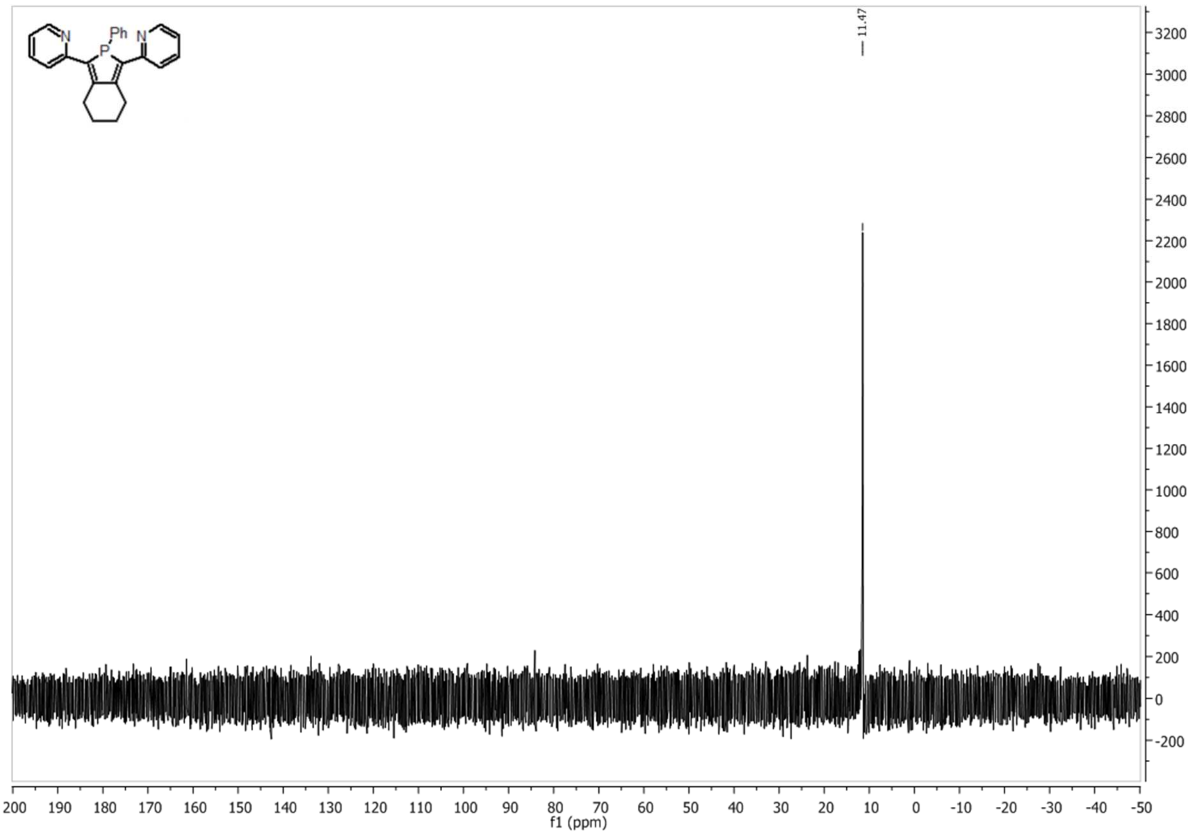
Ph
P
N
11.47
f1 (ppm)

Synthesis of Thienyl-phosphole(3a): 2,2'-octa-1,7-diyne-1,8-diyldithiophene (540 mg, 2.0 mmol) and Cp_2ZrCl_2 (584 mg, 1.40 mmol) were dried for 1h. THF, 30 ml, was added and the solution was cooled to -78 ^{0}C. *n*-BuLi 2.5 M in hexane (1.6 ml, 4.0 mmol) was added at this temperature and the solution turned dark red upon warming to room temperature. The solution was stirred at room temperature for 16 h. The solution was cooled to -78 ^{0}C and $PhPCl_2$ (0.30 ml, 2.1 mmol) was added. The solution was stirred for 4 h and filtered through alumina. The solution was concentrated, redissolved in CH_2Cl_2 and an excess of sulfur was added. The resulting slurry was stirred overnight, filtered and concentrated. Column chromatography with 20 % EtOAc in heptane as eluent yields phosphole-thiophene **3a** as an orange solid. R_f = 0.4. 332 mg (0.62 mmol, 40 %). ^{31}P-NMR ($CDCl_3$, 101 MHz): δ 52.32. ^{1}H-NMR ($CDCl_3$, 300 MHz): δ 1.89 (m, 4H, CH_2), 2.94 (m, 4H, CH_2), 6.96 (dd, *J*= 5.1 Hz, 3.8 Hz, 2H, CH, thienyl), 7.30 (m, *J*= 5.0 Hz, 2.5 Hz, 2H, CH, thienyl), 7.38 (m, 2H, CH, thienyl) overlapping with 7.43 (m, 3H, phenyl), 7.90 (m, 2H, phenyl). ^{13}C-NMR ($CDCl_3$, 75.5 MHz): δ 22.4 (s, C=CCH$_2$*C*H$_2$), 29.1 (d, $J_{P,C}$ = 13.3 Hz, C=C*C*H$_2$CH$_2$), 126.7 (s, thienyl), 127.3 (s, thienyl), 127.7 (d, $J_{P,C}$= 5.5 Hz, thienyl), 128.5 (d, $J_{P,C}$= 83.7 Hz, P*C*=C), 128.8 (d, $J_{P,C}$= 74.1 Hz, ipso-C, phenyl), 128.9 (d, $J_{P,C}$= 12.5 Hz, *m*-C phenyl), 130.7 (d, $J_{P,C}$= 11.7 Hz, ortho-C phenyl), 132.0 (d, $J_{P,C}$= 3.1 Hz, *p*-C phenyl), 134.6 (d, $J_{P,C}$= 17.4 Hz, thienyl), 145.2 (d, $J_{P,C}$= 21.2 Hz, P=C-*C*).

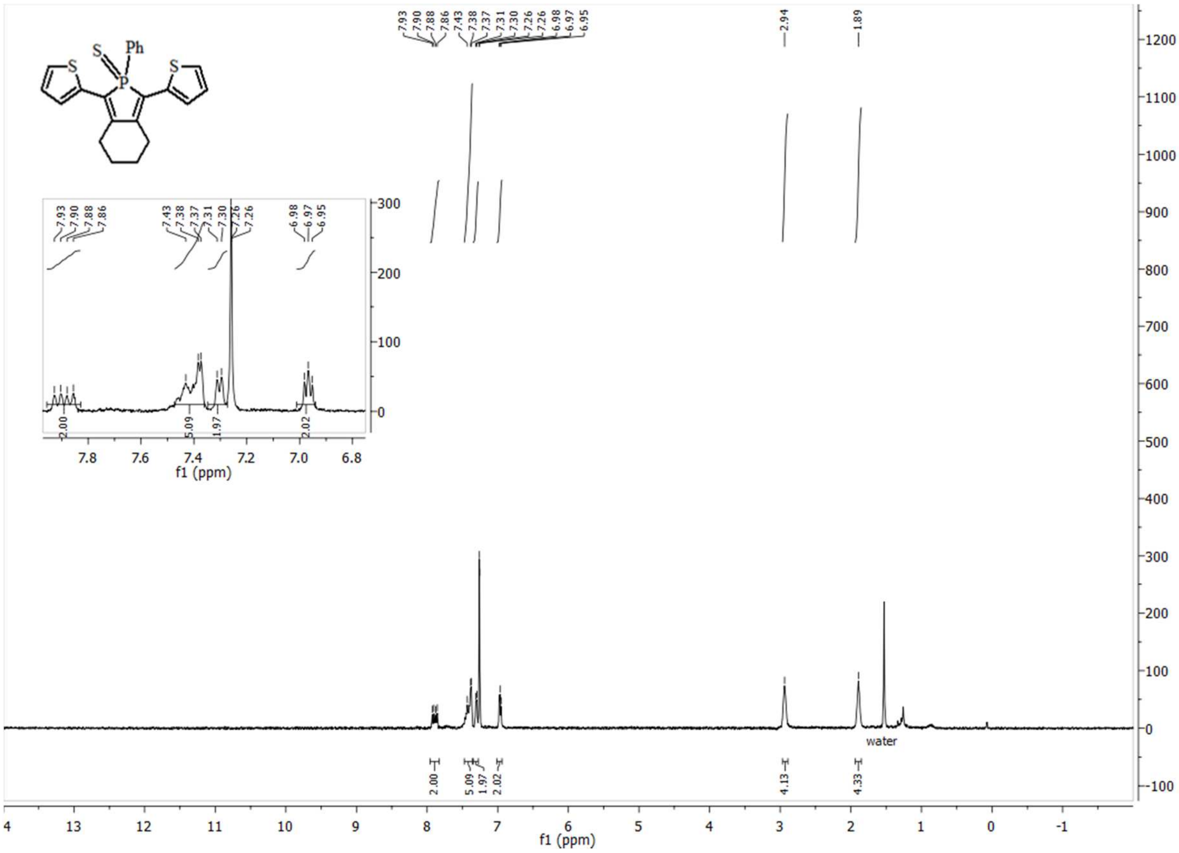

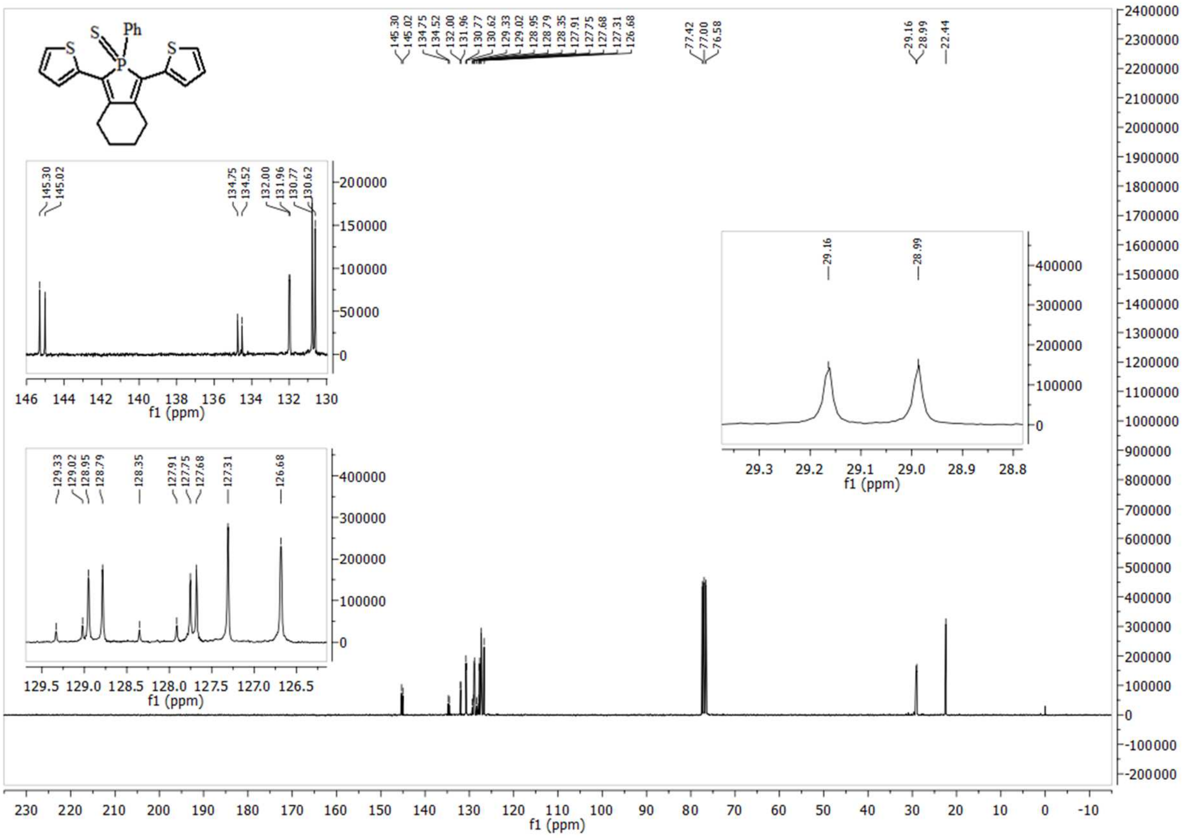
145.30 145.02 134.75 134.52 132.00 131.96 130.77 130.62 129.33 129.02 128.95 128.79 128.35 127.91 127.75 127.68 127.31 126.68
77.42 77.00 76.58
29.16 28.99
22.44
f1 (ppm)

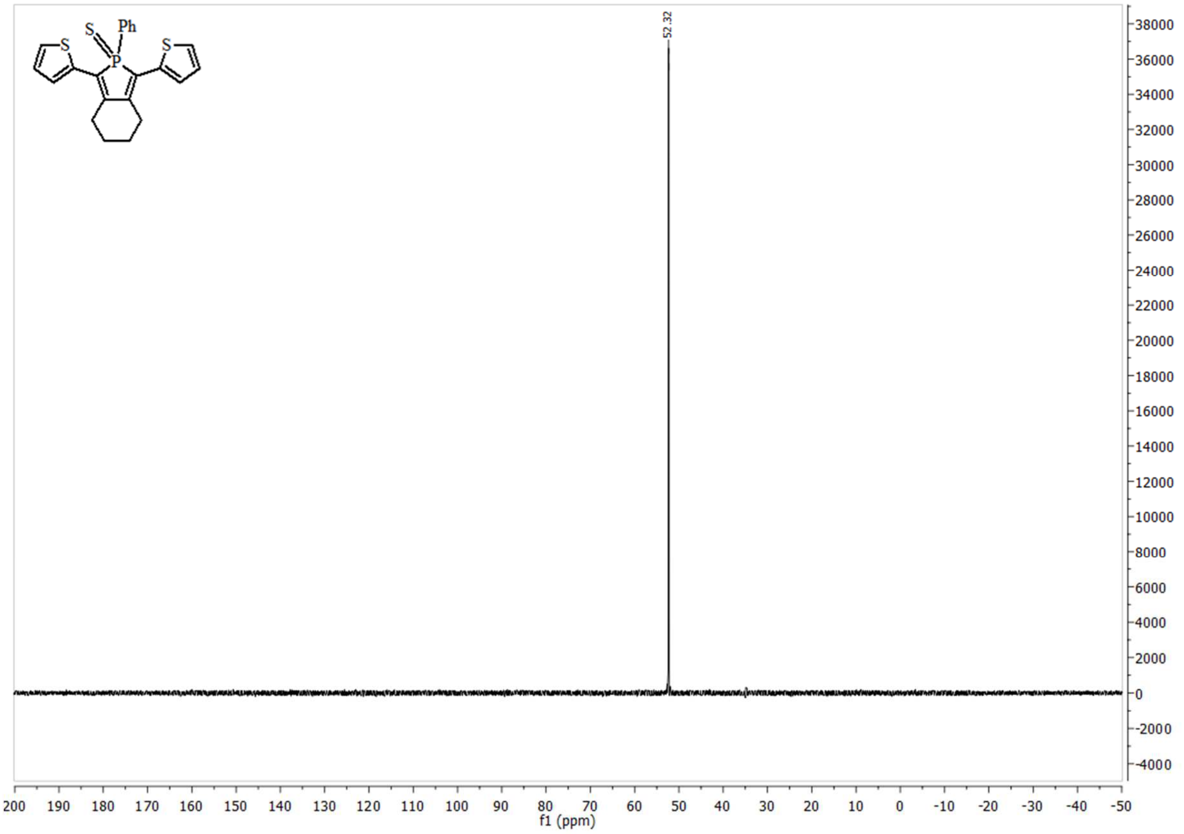
52.32
f1 (ppm)

Synthesis of Pyridyl-phosphole (3b): Phosphole **2b** (86 mg, 0.23 mmol) was dissolved in CH_2Cl_2 and sulfur (32 mg, 1 mmol) was added. The resulting slurry was stirred overnight, filtered and concentrated. Column chromatography in Et_2O yields **3b** as an orange to yellow solid. R_f = 0.8, 88 mg (0.22 mmol, 96 %). ^{31}P-NMR ($CDCl_3$, 101 MHz): δ 52.42 ^{1}H-NMR ($CDCl_3$, 300 MHz): δ 1.81 (m, 4H, CH_2), 3.14 (m, 2H, CH_2) 3.32 (m, 2H, CH_2), 7.08 (ddd, J= 7.7 Hz, 4.7 Hz, 0.8 Hz, 2H, CH, pyridyl), 7.39 (m, 3H, phenyl), 7.56 (td, 2H, J= 7.7 Hz, 1.9 Hz, pyridyl), 7.83 (dd, 2H, J= 8.0 Hz, 0.8 Hz, pyridyl), 7.90 (m, 2H, phenyl), 8.57 (m, 2H, pyridyl). ^{13}C-NMR ($CDCl_3$, 75.5 MHz): δ 22.4 (s, C=CCH_2CH_2), 29.0 (d, $J_{P,C}$ = 12.7 Hz, C=CCH_2CH_2), 122.0 (s, pyridyl), 124.4 (d, $J_{P,C}$= 2.8 Hz, pyridyl), 128.6 (d, $J_{P,C}$= 75.8 Hz,ipso-C Ph), 128.8 (d, $J_{P,C}$= 12.6 Hz, phenyl), 131.7 (d, $J_{P,C}$= 3.0 Hz, phenyl), 132.8 (d, $J_{P,C}$= 83.0 Hz, P=C-C), 135.9 (s, pyridyl), 149.3 (d, $J_{P,C}$= 1.3 Hz, pyridyl), 152.1 (d, $J_{P,C}$= 17.0 Hz, P=C-C), 152.9 (d, $J_{P,C}$= 20.6 Hz, pyridyl).

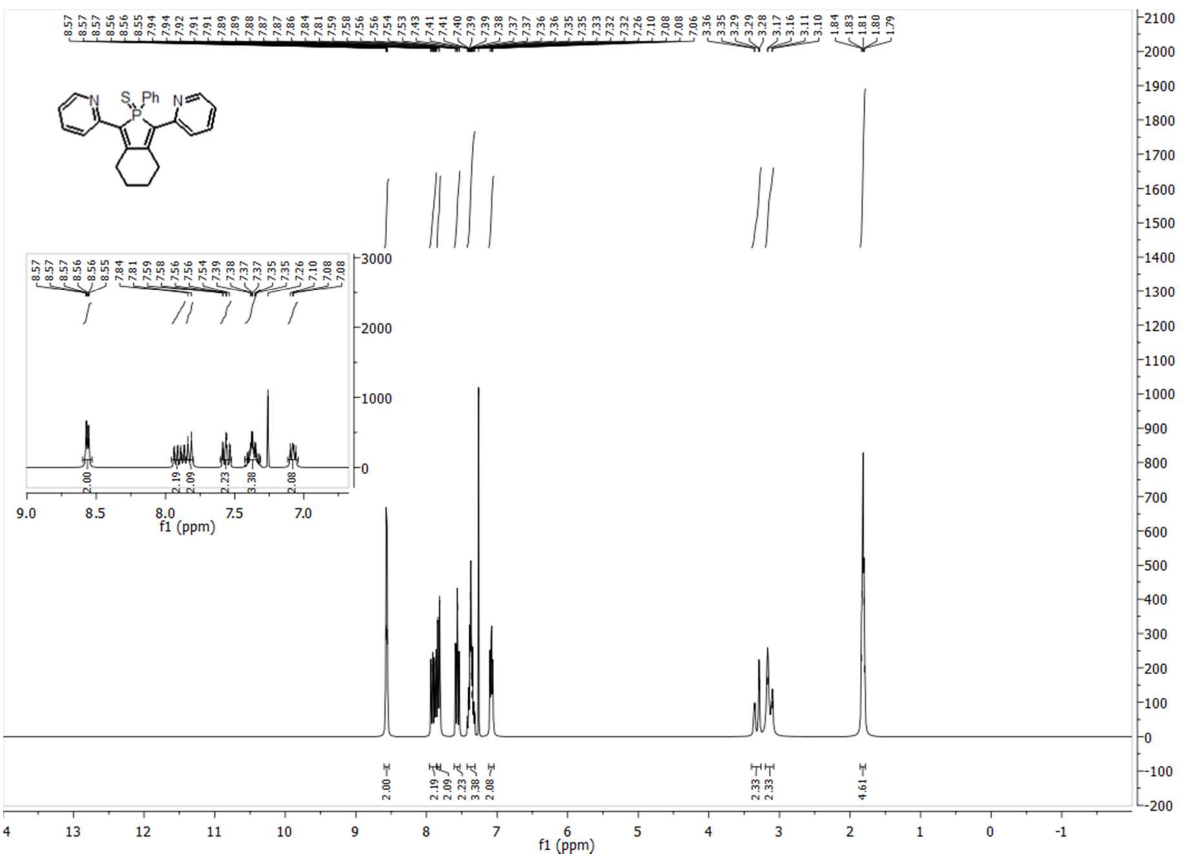

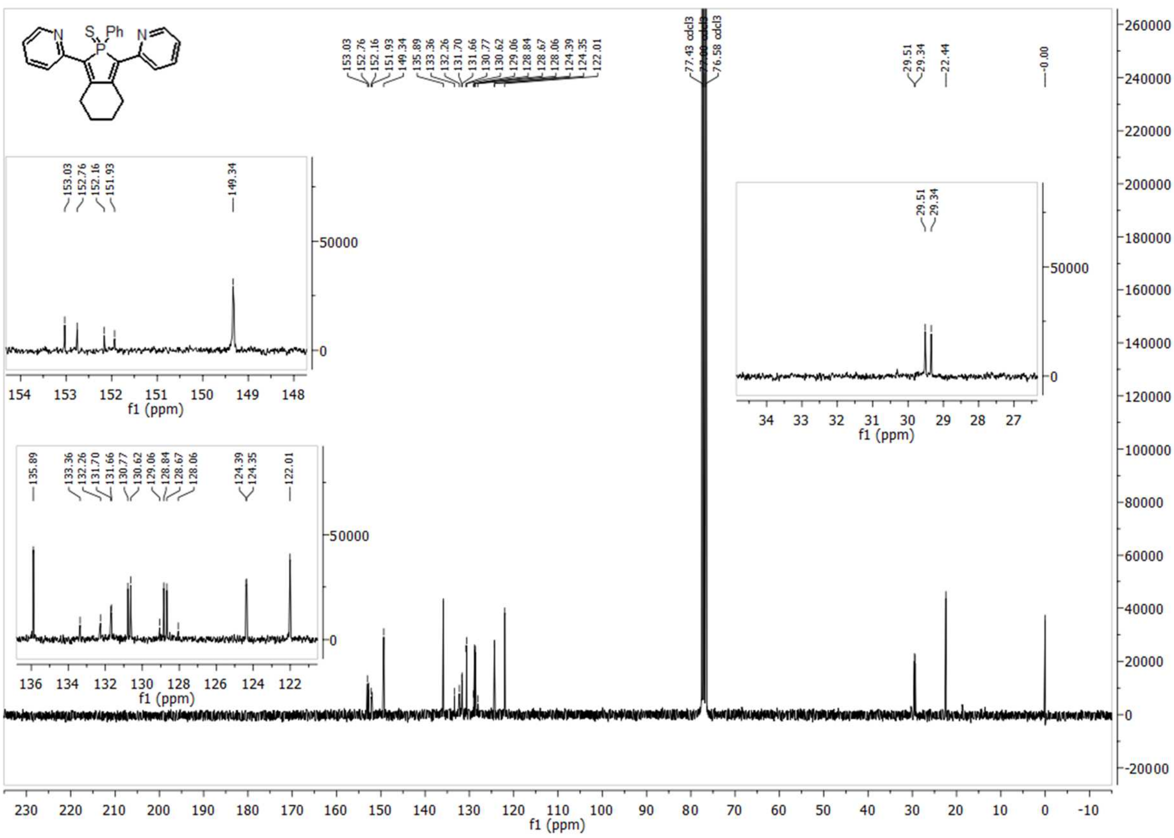
S
Ph
P
N
N
153.03
152.76
152.16
151.93
149.34
135.89
133.36
132.26
131.70
131.66
130.77
130.62
129.06
128.84
128.67
128.06
124.39
124.35
122.01
77.43 cdcl3
77.00 cdcl3
76.58 cdcl3
29.51
29.34
22.44
0.00
f1 (ppm)

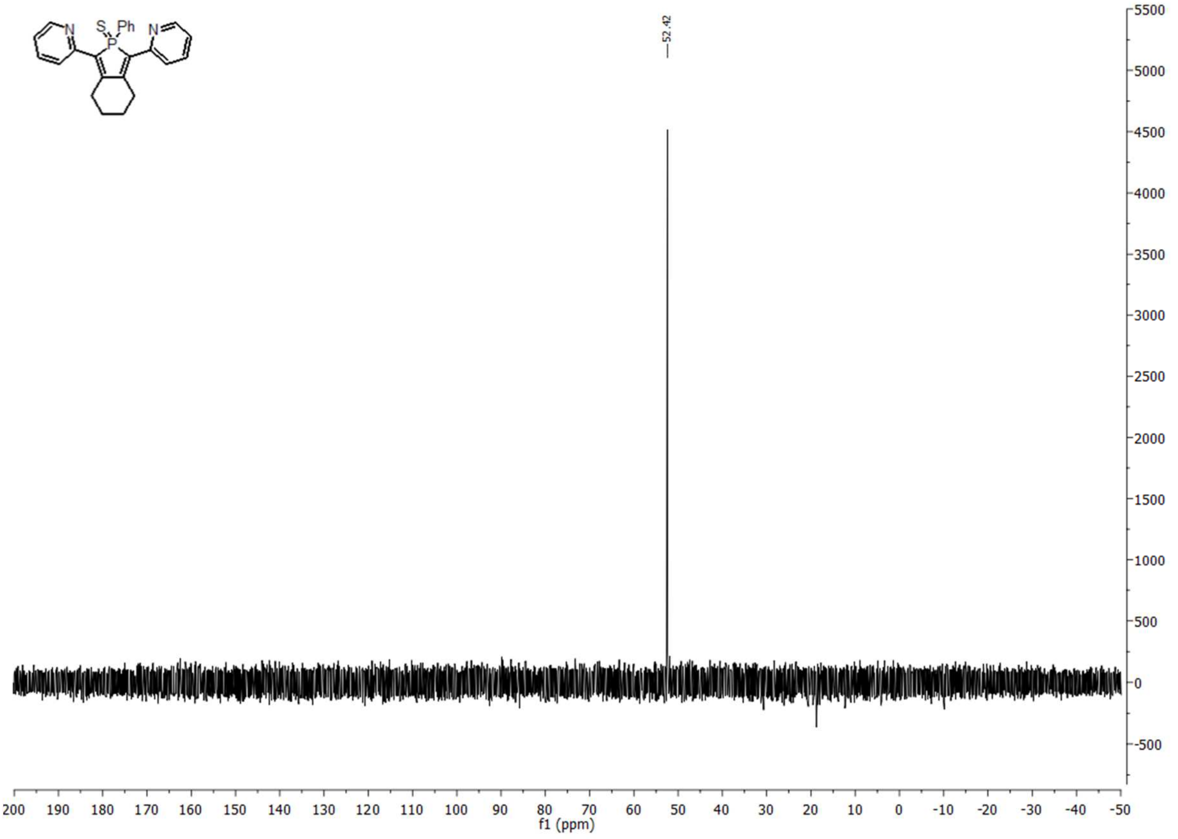
S
Ph
P
N
N
52.42
f1 (ppm)

4. References

Hay, C., Hissler, M., Fischmeister, C., Rault-Berthelot, J., Toupet, L., Nyulászi, L., and Réau, R. (2001) Phosphole-containing pi-conjugated systems: from model molecules to polymer films on electrodes. *Chem. Euro. J.* 7, 4222-4236. doi: 10.1002/1521-3765(20011001)

Fadhel, O., Gras, M., Lemaitre, N., Deborde, V., Hissler, M., Geffroy, B., and Réau, R. (2009) Tunable Organophosphorus Dopants for Bright White Organic Light-Emitting Diodes with Simple Structures. *Adv. Mater.* 21, 1261-1265. DOI: 10.1002/adma.200801913
